# Association of cerebral microdialysis monitoring with clinical outcomes after traumatic brain injury; A systematic review

**DOI:** 10.1016/j.bas.2026.106659

**Published:** 2026-09-02

**Authors:** Cansu Rehber, Sara Turella, Wilco C. Peul, Raimund Helbok, Thomas A. van Essen, Jeroen T.J.M. van Dijck, SOPRANI Collaborators, Alan Urban, Gabriel Montaldo, Eloïse Baud, Dick Moberg, Geert Meyfroidt, Fadime Tokmak, Bart Depreitere, Fabio Gonçalves, Elle Scheijen, Jens P. Dreier, Malaika Mohammad, Coline L. Lemale, Samira Saadoun, Marios C. Papadopoulos, Chibuzor Love Ilochonwu, Peter Smielewski, Wenhao Xu, Raimund Helbok, Sara Turella, Ewout W. Steyerberg, Nicole S. Erler, Eugenia Driusso, Aneeta Chacko, Wilco Peul, Thomas van Essen, Jeroen van Dijck, Cansu Rehber

**Affiliations:** e Flemish Institute for Biotechnology (VIB), KU Leuven, Leuven, Belgium; f Moberg Analytics, Inc., Philadelphia, USA; g Laboratory of Intensive Care Medicine, Department of Cellular and Molecular Medicine, KU Leuven, Leuven, Belgium; h Research Group Experimental Neurosurgery and Neuroanatomy, Department of Neurosciences, KU Leuven, Leuven, Belgium; i Centre for Stroke Research Berlin, Charité – Universitätsmedizin Berlin, Corporate Member of Freie Universität Berlin, Humboldt-Universität zu Berlin, and Berlin Institute of Health, Berlin, Germany; j Department of Neurosurgery, St George's University Hospitals NHS Foundation Trust, London, United Kingdom; k Brain Physics Laboratory, Department of Clinical Neurosciences, University of Cambridge, Cambridge, United Kingdom; l Department of Neurology, Kepler University Hospital, and Clinical Research Institute for Neuroscience, Johannes Kepler University Linz, Linz, Austria; m Julius Center for Health Sciences and Primary Care, University Medical Center Utrecht, Utrecht, the Netherlands; n Department of Neurosurgery, University Neurosurgical Center Holland, Leiden University Medical Center, Haaglanden Medical Center, Haga Teaching Hospital, Leiden and The Hague, the Netherlands; a Department of Neurosurgery, University Neurosurgical Center Holland, Leiden University Medical Center, Haaglanden Medical Center, Haga Teaching Hospital, Leiden and The Hague, the Netherlands; b Leiden University Medical Center, Leiden, the Netherlands; c Department of Neurology, Kepler University Hospital, Johannes Kepler University Linz, Linz, Austria; d Clinical Research Institute for Neuroscience, Johannes Kepler University Linz, Linz, Austria

**Keywords:** Traumatic brain injury, Head injury, Cerebral microdialysis, Clinical outcome, Secondary brain injury

## Abstract

**Objective:**

Cerebral microdialysis (CMD) enables bedside monitoring of cerebral metabolism after traumatic brain injury (TBI). This systematic review evaluates associations between CMD-derived analytes and mortality and functional outcome in adults with moderate-to-severe TBI.

**Methods:**

A systematic review was conducted of adult patients with moderate-to-severe TBI (Glasgow Coma Scale ≤12) undergoing CMD monitoring of glucose, lactate, pyruvate, lactate/pyruvate ratio (LPR), glutamate, and/or glycerol. Prespecified outcomes were mortality and functional outcome (Glasgow Outcome Scale, Glasgow Outcome Scale-Extended or modified Rankin Scale). Due to substantial between-study heterogeneity, findings were synthesized qualitatively.

**Results:**

The database search identified 1342 records; 33 studies met inclusion criteria. CMD-glucose and CMD-lactate were the most commonly measure analytes (26 studies each), and the least was CMD-glycerol (12 studies). 9 studies have analyzed as a combined biochemical pattern or multimodal modality in relation to outcome. Higher CMD-glucose levels were mostly associated with better outcomes, whereas lower CMD-glucose and elevated CMD- LPR, CMD-glutamate and CMD-glycerol levels were associated with mortality or unfavorable functional outcome. However, analyte thresholds, probe locations, monitoring windows and outcome definitions varied widely and adjusted prognostic estimates were rarely reported. Certainty of evidence was predominantly low to very low.

**Conclusion:**

CMD biomarkers show directional associations with outcome after moderate-to-severe TBI, but the evidence is heterogeneous and of low certainty, insufficient to support routine use of CMD analytes for prognostic stratification. Standardization of analyte thresholds, probe location, monitoring windows is needed before their prognostic value can be established.

## Highlights

• 33 studies assessed CMD analytes as prognostic markers in moderate-to-severe TBI.
• Reported associations were directionally consistent but of low to very low certainty.
• Higher glucose trended to better outcome; higher LPR, glutamate, glycerol to worse.
• Thresholds, probe sites, monitoring windows, and outcome measures varied widely.
• Evidence is insufficient to support routine prognostic use.

## 1 Introduction

Traumatic brain injury (TBI) is a major public health problem worldwide and remains a leading cause of death and long-term disability across all age groups. Survivors frequently experience persistent neurological, cognitive, and emotional impairments that substantially affect functional outcome and quality of life (Maas et al., 2008; Roozenbeek et al., 2013; Steyerberg et al., 2008). Accurate early prognostication is therefore essential to guide clinical decision-making, stratify patients for targeted therapies, and inform families. Traditionally, prognosis in TBI has relied on baseline clinical and radiological characteristics reflecting injury severity, including neurological examination, the Glasgow Coma Scale (GCS), computed tomography (CT) findings, and intracranial pressure (ICP) monitoring (Hawryluk et al., 2020; Collaborators, 2008; Gao and Zheng, 2015).

Secondary brain injury is a dynamic and potentially modifiable process characterized by metabolic, inflammatory, and biochemical disturbances initiated by the primary insult (Hillered et al., 2014). These secondary mechanisms play a critical role in determining long-term outcome. Neurointensive and neurosurgical management strategies aim to prevent or mitigate secondary injury through physiological optimization, commonly using ICP and cerebral perfusion pressure (CPP) guided therapy (Chesnut et al., 2020; Bögli et al., 2025; White and Venkatesh, 2008; van Erp et al., 2023). However, conventional monitoring primarily reflects global cerebral physiology and may fail to capture evolving regional metabolic disturbances at the cellular level.

Cerebral microdialysis (CMD) is an advanced neuromonitoring technique that enables continuous sampling of extracellular metabolites from the brain parenchyma via a semipermeable catheter (Majdan et al., 2017). Key analytes including glucose, lactate, pyruvate, the lactate/pyruvate (LP) ratio, glutamate, and glycerol, provide real-time insights into cerebral energy metabolism, ischemia, cellular membrane breakdown, and excitotoxicity (Zeiler et al., 2017a; Paraforou et al., 2011). CMD is increasingly incorporated into multimodal neuromonitoring protocols in specialized neurocritical care units, alongside ICP, CPP, and brain tissue oxygenation monitoring (Carteron et al., 2017).

Despite this growing use, the clinical value of CMD as a prognostic tool remains incompletely defined. Abnormal CMD-derived metabolic patterns have been linked to mortality and unfavorable neurological states, but it is unclear whether CMD provides independent and robust prediction of long-term functional outcome beyond established clinical, radiological, and physiological prognostic markers (Ståhl et al., 2001). This uncertainty represents a critical gap in the evidence base and underpins the rationale for the present study. Our primary aim is to evaluate the prognostic significance of individual CMD analytes and combined metabolic patterns for mortality and functional outcome after moderate-to-severe TBI, and to rate the certainty of this evidence.

## 2 Methods

This review was designed and reported as a prognostic systematic review, treating CMD biomarkers as prognostic factors rather than therapeutic interventions. CMD analytes were considered exposures, and mortality and functional outcome were considered prognostic outcomes. Our objective was to summarize reported CMD-outcome associations and to appraise the quality and certainty of the prognostic evidence.

The systematic review was pre-registered in PROSPERO (registration number: CRD42024607691) and data were reported using the PRISMA 2020 Guidelines (Page et al., 2021).

### 2.1 Search strategy and article selection

**Fig. 1:**
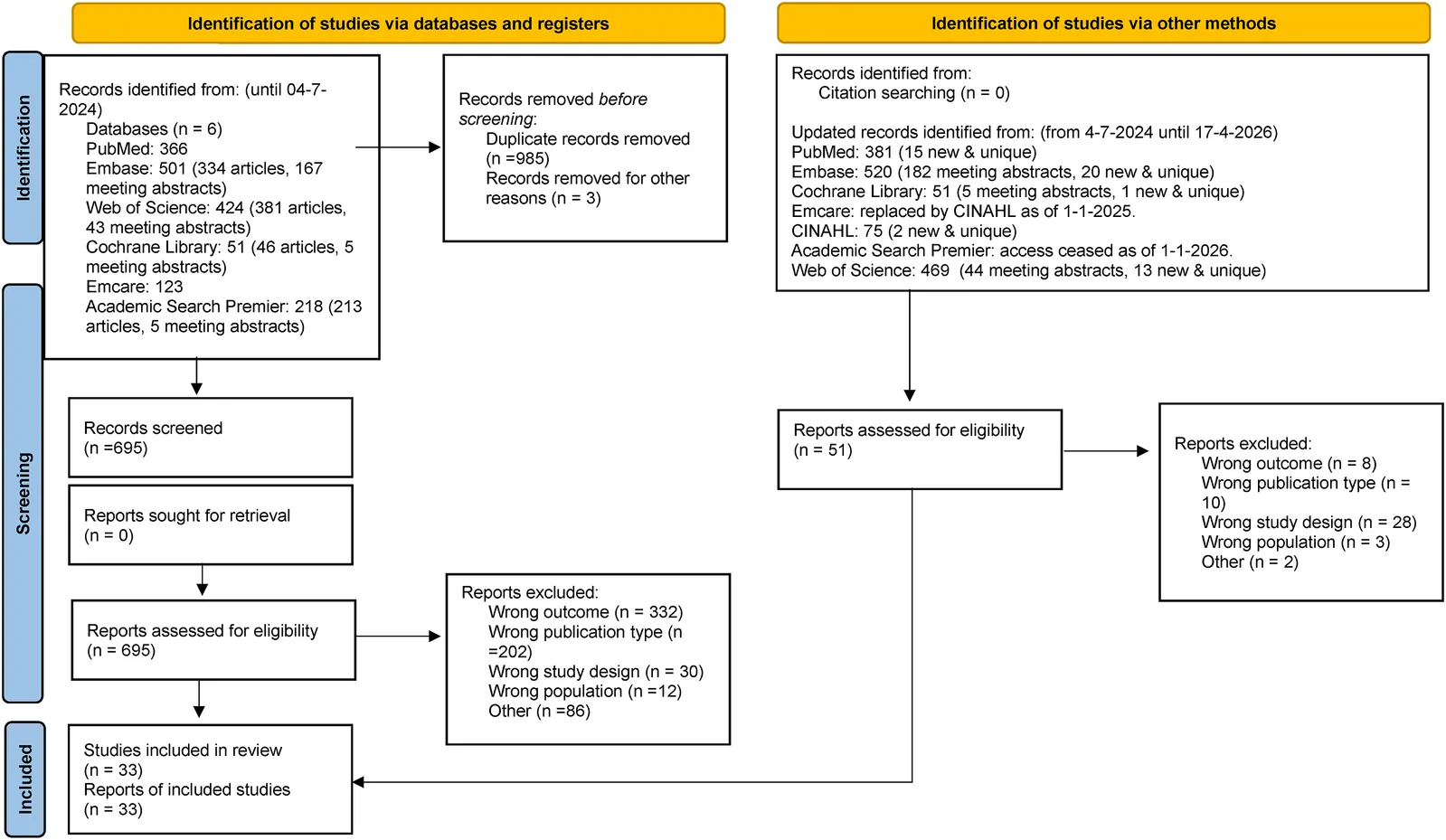
PRISMA flow diagram (Page et al., 2021).

The initial database search (PubMed, Embase, Web of Science, Cochrane Library, Emcare and Academic Search Premier) until 4 July 2024 identified 1683 records. After removal of 985 duplicates and 3 records excluded for other reasons, 695 unique records remained. The updated search to 17 April 2026 identified 51 additional unique records, yielding 746 records for screening. The large majority were excluded at title and abstract or full-text assessment. The principal reasons for exclusion were outcome not reported, that is, no mortality or functional outcome data (n = 340); ineligible publication type, comprising conference abstracts, narrative reviews, editorials, commentaries, and case reports (n = 212); other reasons (n = 88); ineligible study design (n = 58); and a population not restricted to moderate or severe TBI (n = 15). Thirty-three studies met all inclusion criteria (32 observational, 1 randomized) (Search strategy can be found in Appendix 1.) (Fig. 1).

Two reviewers independently reviewed titles and abstracts followed by an in-depth full text screening for eligibility. In case of discrepancies, a third independent reviewer was consulted. All analyses, and article inclusion decisions were managed using the Rayyan system (Ouzzani et al., 2016).

### 2.2 Inclusion and exclusion criteria

We included studies of adult patients with moderate-to-severe TBI (GCS ≤12) in which CMD was applied to monitor at least one of the following metabolites: glucose, lactate, pyruvate, glutamate, and/or glycerol. Outcomes of interest included functional recovery, assessed using validated scales such as the Glasgow Outcome Scale (GOS), Glasgow Outcome Scale Extended (GOSE), and modified Rankin Scale (mRS) scores, as well as mortality. Pediatric population was excluded. Functional outcomes were considered only when reported at a clearly defined time point (e.g., discharge, 3 months, or 6 months post-injury), as outcomes assessed at inconsistent or undefined intervals were considered insufficient.

Regarding study design, only full-text peer-reviewed publications were eligible for inclusion. Conference and meeting abstracts were identified and evaluated during the screening process; however, they were ultimately excluded from the final synthesis. This decision was based on the likelihood that abstracts represent preliminary or duplicate findings from studies already captured in the full-text literature, as well as their inherently limited reporting of methodology, which introduces a high risk of bias and precludes adequate quality assessment.

In studies enrolling mixed patient populations (e.g., combining TBI patients with other neurological or neurosurgical diagnoses), data were eligible for inclusion only when results for the moderate-to-severe TBI subgroup (GCS ≤12) were reported separately.

### 2.3 Data extraction

Extracted data included year of publication, study design, sample size, patient characteristics, inclusion criteria, primary objectives, CMD analytes monitored, and outcome measurements of interest (GOS, GOSE, mRS, mortality and morbidity), alongside study results and conclusions. Where studies reported outcomes at multiple time points, all available time points were extracted and recorded in the data extraction table. For studies with mixed populations, only subgroup-specific data pertaining to moderate-to-severe TBI patients (GCS ≤12) were extracted; studies in which such data could not be disaggregated were excluded. All key data fields were retrievable from the included studies, and no author correspondence was necessary.

### 2.4 Quality assessment

Because CMD analytes are measured rather than assigned or manipulated, they function as prognostic factors rather than interventions. Standard risk-of-bias tools are designed primarily for interventional research and are therefore not directly applicable to this study design. Accordingly, we assessed risk of bias using the QUIPS(Hayden et al., 2013) tool. Two reviewers independently rated risk of bias at the study level; disagreements were resolved through discussion or third-party adjudication.

The certainty of evidence for each CMD analyte was assessed using the GRADE (Guyatt et al., 2008) approach with prognostic-factor-specific guidance (Foroutan et al., 2020). Because prognostic factor questions are typically addressed using observational designs, bodies of evidence were initially considered high certainty, and were then rated down based on five domains: risk of bias, inconsistency, indirectness, imprecision, and publication bias. Downgrades of one or two levels were applied per domain as appropriate. Final certainty ratings were classified as high, moderate, low, or very low.

### 2.5 Data analysis

Neurochemical markers (glucose, lactate, pyruvate, glutamate, and glycerol) were analyzed in relation to functional outcomes (GOS, GOSE, mRS), mortality, and morbidity. Given the heterogeneity in study designs, patient populations, CMD monitoring protocols, and outcome reporting across the included literature, a meta-analysis was not performed. Instead, a qualitative narrative synthesis was conducted, grouping findings by neurochemical marker, TBI severity, and outcome measure. A combined biochemical pattern was defined as the joint analysis of two or more analytes as a composite index or metabolic state in relation to outcome, distinct from the concurrent measurement of multiple analytes reported separately.

The effect of interest was the association between CMD analyte levels, including absolute values and derived ratios, and functional outcome at the study-defined follow-up point. Where reported, directionality of association and any threshold values proposed by study authors were documented.

## 3 Results

### 3.1 Study selection and characteristics

Of 746 unique records identified, 33 studies met the inclusion criteria (Paraforou et al., 2011; Chamoun et al., 2010; Clausen et al., 2005a, 2005b; Eiden et al., 2019; Goodman et al., 1999; Gopinath et al., 2000; Hägglund et al., 2022; Hejčl et al., 2011; Hlatky et al., 2007; Ho et al., 2008; Igarashi et al., 2017; Karathanou et al., 2011; Koura et al., 1998; Li et al., 2008; Low et al., 2009; Marini et al., 2022; Mazzeo et al., 2006; Nelson et al., 2011; Nordström et al., 2016; Oddo et al., 2008; Olivecrona et al., 2009; Peerdeman et al., 2003; Rajagopalan et al., 2022; Reinert et al., 2007; Sanchez et al., 2013; Sánchez-Porras et al., 2012; Stein et al., 2012; Thelin et al., 2014; Vespa et al., 2003, 2006; Zauner et al., 1997; Gupta et al., 2017) (32 observational cohorts and 1 randomized controlled trial (Olivecrona et al., 2009)). Twelve studies were conducted in Europe and twelve in North America, with a further five in Asia and four conducted as European-North American collaborations. Sample sizes ranged from 12 to 213 patients, and most studies (26/33) enrolled patients with severe TBI (GCS 3-8) (Paraforou et al., 2011; Chamoun et al., 2010; Clausen et al., 2005a, 2005b; Eiden et al., 2019; Gopinath et al., 2000; Hägglund et al., 2022; Hejčl et al., 2011; Ho et al., 2008; Karathanou et al., 2011; Koura et al., 1998; Li et al., 2008; Low et al., 2009; Marini et al., 2022; Mazzeo et al., 2006; Nelson et al., 2011; Nordström et al., 2016; Oddo et al., 2008; Olivecrona et al., 2009; Peerdeman et al., 2003; Reinert et al., 2007; Sánchez-Porras et al., 2012; Thelin et al., 2014; Vespa et al., 2003; Zauner et al., 1997; Gupta et al., 2017), whereas three studies included patients with moderate-to-severe TBI (GCS 3-12) (Ho et al., 2008; Stein et al., 2012; Vespa et al., 2006) (Table 1).

**Table 1:** Characteristics of included studies.

Author (Year)	Article Title	Type of Article	Region/Country	Study Design	Sample Size	Patient characteristics	Inclusion Criteria	Primary Objectives	CMD* analytes	CMD Probe Placement	Outcome measurements	Results
Chamoun et al. (2010)	Role of extracellular glutamate measured by cerebral microdialysis in severe traumatic brain injury	Journal Article	United States	Prospective Observational Cohort	165	Severe TBI: GCS score ≤ 8	TBI, a blunt mechanism of head trauma, and a GCS score ≤ 8 on presentation or within 48 h of injury.	Glutamate's impact on the disease's pathogenesis and correlation with the clinical outcomes	Glutamate	Non-lesional brain tissue	- Survival and mortality - GOS at 6 months	Threshold: CMD-glutamate >20 μmol/L Direction: persistently elevated or rising glutamate over 120 h independently associated with worse mortality and functional outcome Mortality effect: 39.6% vs 17.1%, (multivariate p = 0.02) Functional outcome effect: (survivors, GOS 4–5): 20.7% vs 41.2% (multivariate p = 0.03)
Clausen et al. (2005)	Association between elevated brain tissue glycerol levels and poor outcome following severe traumatic brain injury	Journal Article	Portugal, Switzerland, United States	Prospective Observational Cohort	76	Severe TBI: GCS 3–8	Patients with severe closed-head injury (GCS 3–8 on admission or within 12 h postadmission) who had been admitted to the neurosurgical ICU at MCV were included in this study.	association between microdialysate glycerol concentration, brain tissue oxygen levels and clinical outcomes (GOS score at 3 months)	Glycerol	Non-lesional brain tissue, right lobe	GOS at 3 months	CMD-glycerol GOS 4-5: mean 63 ± 10 mmol/L GOS 1-2: 83 ± 15 mmol/L; over the entire monitoring period
Clausen et al. (2005)	Cerebral acid-base homeostasis after severe traumatic brain injury	Journal Article	Germany, Portugal, Switzerland, United States	Prospective Observational Cohort	151	Severe TBI: GCS 3–8	GCS 3–8 at admission or within 12 h after admission, who were admitted to the neuroscience ICU at VCUHS, were included in the study.	association between brain tissue acid-base disturbances (specifically acidosis) and clinical outcomes. (GOS scores at 3 months)	Lactate	Non-lesional brain tissue	GOS at 3 months	CMD-lactate GOS 4-5: 785 ± 119 mmol/L GOS 1-2: 1051 ± 118 mmol/L; overall cohort mean 976 ± 71 mmol/L Direction: higher lactate associated with poor outcome
Eiden et al. (2019)	Discovery and validation of temporal patterns involved in human brain ketometabolism in cerebral microdialysis fluids of traumatic brain injury patients	Journal Article	Switzerland, United Kingdom	Prospective observational cohort	38	Severe TBI: GCS < 9 and abnormal CT scan (defined by the presence of intracranial lesions [contusions, hematoma)	Severe TBI patients with a post-resuscitation GCS <9 and an abnormal CT scan (defined by the presence of intracranial lesions [contusions, hematoma]), who underwent brain monitoring with CMD, ICP and PbtO2 as part of standard patient care.	clinically relevant metabolic patterns at the acute post-TBI critical care phase	Lactate, Pyruvate, Glucose, Glutamate	Non-lesional brain tissue	GOS at 6 months	State A: characterized by higher CMD-lactate, pyruvate, and glucose with lower glutamate and ICP, was associated with better 6-month outcome compared with “State B″ CMD-lactate: Higher in state A p < 0.0001 CMD-pyruvate: Higher in state A p < 0.0001 CMD-glucose: Higher in state A p < 0.0001 CMD-glutamate: Lower in state A p < 0.0001
Goodman et al. (1999)	Extracellular lactate and glucose alterations in the brain after head injury measured by microdialysis	Journal Article	United States	Prospective, nonrandomized, clinical study.	126	Severe TBI: GCS <9	Individuals >18 years of age with head injury necessitating craniotomy or placement of ICP monitoring sensors.	To study cerebral glucose and lactate metabolism in head-injured patients using microdialysis	Glucose, Lactate	Non-lesional brain tissue	Survival	CMD-lactate: patients who died: 0.68 (0.45–1.05) patients who survived: 0.85 (0.57–1.66) μmol/mL; p = 0.04 CMD-L/G ratio: patients who died: 2.7 (1.5–4.9) patients who survived 6.7 (1.6–13.0); p = 0.004 CMD-glucose: NS CMD glucose = 0 (% of samples) Higher proportion of zero-glucose samples in patients who died 0.9% (0–8.2%) vs 6.5% (0–16.7%); p = 0.013
Gopinath et al. (2000)	Extracellular glutamate and aspartate in head injured patients	Journal Article	United States	Prospective Observational Cohort	86	Severe TBI: GCS ≤ 8	patients who had a GCS ≤ 8 on admission or who deteriorated to a GCS ≤ 8 within 48 h of admission	Examine clinical factors that are related to the release of excitotary aminoacids	Glutamate	Perilesional brain tissue	GOS	CMD-glutamate: median glutamate 7.4 μM (IQR 3.6–18.8) patients that died: 29.5 μM (9.1–147.0); GOS 4–5: 7.2 μM (4.4–12.0); GOS 2–3: 7.6 μM (5.7–17.2); p < 0.001
Gupta et al. (2017)	Detection of metabolic pattern following decompressive craniectomy in severe traumatic brain injury: A microdialysis study	Journal Article	India	Prospective Observational Cohort	41	Severe TBI: post-resuscitation GCS ≤ 8 (undergoing decompressive craniectomy)	Adult patients with severe TBI (post-resuscitation GCS ≤ 8) requiring decompressive craniectomy for refractory intracranial hypertension (ICP > 20 mmHg for > 30 min), presenting with cerebral contusions or acute subdural hematoma within 24 h of injury.	To detect mitochondrial dysfunction and ischemia by CMD and their relationship with outcome following decompressive craniectomy	Glucose, Lactate, Pyruvate, Glutamate, Glycerol, LP ratio	Predominantly on perilesional tissue/non-eloquent tissue after SDH evacuation	GOS at 3 and 6 months, dichotomized (GOS 4–5 favorable; GOS ≤ 3 unfavorable); mortality	Metabolic states defined as mitochondrial dysfunction (LPR > 25, pyruvate > 70 μmol/L) and ischemia (LPR > 25, pyruvate < 70 μmol/L). 26/41 (63.4%) had favorable and 15 (36.6%) unfavorable outcome at 6 months; 11 patients died. The unfavorable group spent significantly more monitoring time in mitochondrial dysfunction (65.9% vs 55.9%, p < 0.001) and ischemia (13.9% vs 7.2%, p < 0.001). On ordinal regression (adjusted for age and post-resuscitation GCS, both non-significant), a 10% increase in time in ischemia doubled the odds of poor outcome (OR 2.04, 95% CI 1.12–3.73) and a 10% increase in mitochondrial dysfunction increased the odds (OR 1.48, 95% CI 1.07–2.04). MD-detected ischemia versus mitochondrial dysfunction showed lower cerebral glucose (0.84 vs 1.98 mmol/L), higher glutamate (59.6 vs 32.9 μmol/L) and higher LPR (139.6 vs 49.6) (all p < 0.001).
Hägglund et al. (2022)	Correlation of Cerebral and Subcutaneous Glycerol in Severe Traumatic Brain Injury and Association with Tissue Damage	Journal Article	Sweden	Retrospective analysis of prospectively collected data	42	Severe TBI: GCS ≤8	Verified severe traumatic head injury, a GCS of 8 or less at intubation and sedation, age between 15 and 70 years, a first measured CPP of 10 mm Hg or higher, and arrival at our unit within 24 h of trauma.	investigate whether there was a correlation between brain and subcutaneous glycerol levels and whether the ratio of interstitial glycerol in the brain and subcutaneous tissue (glycerolbrain/sc) was associated with tissue damage in the brain	Glycerol	Catheter A: perilesional tissue Catheter B: other hemisphere	GOSE at 6 months	Negative glycerol (brain/sc) correlation group vs positive correlation group: No significant difference. Median 4 (range 1–8) vs 5 (1–7); not significant (Wilcoxon rank-sum)
Hejčl et al. (2011)	Elevated Intracranial Pressure, Low Cerebral Perfusion Pressure, and Impaired Brain Metabolism Correlate with Fatal Outcome After Severe Brain Injury	Journal Article	Czech Republic	Prospective Observational Cohort	20	Severe TBI: GCS <9	not provided	predictive value of monitoring brain oxygenation and metabolism on clinical outcome	Glucose, Glycerol, Lactate and Pyruvate	Perilesional brain tissue (biovhemical penumbra)	GOS at 6 months	CMD-glucose patients who survived: 0.96 ± 0.13 mmol/l; patients who died: 1.09 ± 0.19 mmol/l p > 0.05, ns CMD-L/P ratio patients who survived: 37.36 ± 3.44 patients who died: 199.26 ± 87.97 p < 0.05 CMD-glycerol patients who survived: 62.07 ± 12.14 μmol/l, patients who died: 215 ± 46.52 μmol/l, p < 0.05
Hlatky et al. (2007)	Evolution Of Brain Tissue Injury After Evacuation Of Acute Traumatic Subdural Hematomas	Journal Article	United States	prospective Observational Cohort	33	moderate to severe TBI (GCS range 3-10)	those diagnosed with acute traumatic subdural hematoma requiring surgical evacuation	To analyze tissue biochemical patterns in the brain underlying an evacuated acute subdural hematoma	glucose, lactate, pyruvate, and glutamate	Lesional Tissue	GOSE at 6 months	CMD-lactate: delayed traumatic injury (DTI): 4.1 ± 2.3 mmol/L No-DTI: 1.7 ± 0.7 mmol/L; overall group effect p < 0.001 CMD-pyruvate: DTI: 104 ± 47 μmol/L No-DTI: 73 ± 54 μmol/L; overall group effect p < 0.001 Patients with DTI had significantly higher mortality (53% vs 9%, p < 0.002) and higher lactate and pyruvate levels, supporting an association between metabolic disturbance and poor outcome.
Ho et al. (2008)	Cerebral oxygenation, vascular reactivity, and neurochemistry following decompressive craniectomy for severe traumatic brain injury	Journal Article	Singapore	Retrospective Study	16	moderate to severe TBI (GCS range 3-10)	patients with both severe TBI and elevated ICP refractory to medical therapy were included	effect of decompressive craniotomy on ICP levels, neurochemistry and clinical outcomes	glucose, lactate, pyruvate, and glutamate	Perilesional tissue	GOS at 6 months	CMD-lactate: GOS4-5, pre- vs post-DC: Significant reduction, 7 ± 4 → 3 ± 2 mmol/L; p < 0.001 GOS 1-2, pre- vs post-DC: Marginal reduction, 6 ± 2 → 5 ± 2 mmol/L; p = 0.04 CMD-L/P ratio: GOS4-5, pre- vs post-DC: Significant reduction, 137 ± 85 → 40 ± 12; p < 0.001 GOS 1-2, pre- vs post-DC: Marginal reduction, 54 ± 10 → 39 ± 5; p = 0.04 CMD-glutamate: GOS4-5, pre- vs post-DC: Significant normalization, 36 ± 54 → 3 ± 1 mmol/L; p < 0.001 GOS 1-2, pre- vs post-DC: (NS) CMD-glucose: GOS4-5, pre- vs post-DC: 0.6 ± 2 → 1 ± 1 mmol/L; p < 0.001 GOS 1-2, pre- vs post-DC: (NS)
Igarashi et al. (2017)	Relation between extracellular Chemistry and Patient Outcome for Severe Traumatic Brain Injury within the First 24 h: A Microdialysis Study	Journal Article	Japan	Prospective Observational Cohort	30	Severe TBI (GCS ≤8)	Severe TBI associated with focal brain injury who were admitted to the department from 2009 to 2011. Focal brain injury was defined as acute subdural hematoma, acute epidural hematoma, and cerebral contusion without diffuse brain injury.	Relationship between extracellular chemistry in a shorter period and the outcome of patients with focal brain injury	glucose, lactate, pyruvate, glycerol, and glutamate	Lesional (white matter were hematoma was removed)	- Survival and mortality - GOS at discharge (Favorable outcome: good recovery or moderate disability; unfavorable outcome; severe disability, persistent vegetative state or death)	CMD-glycerol and L/P ratio were the strongest 24-h predictors: glycerol 124 vs 808 μmol/L (favorable vs unfavorable, p = 0.002) and 168 vs 1462 μmol/L (survivors vs died, p = 0.002); L/P ratio 31 vs 48 (p = 0.021) and 32 vs 124 (p < 0.001). Glucose, lactate, glutamate, and L/G ratio also differed significantly between survivors and non-survivors (p = 0.035, 0.018, 0.019, 0.004), but not between favorable/unfavorable outcome. Pyruvate showed no significant association.
Karathanou et al. (2011)	Biochemical markers analyzed using microdialysis and traumatic brain injury outcomes	Journal Article	Greece	Prospective Observational Cohort	38	Severe TBI: GCS score ≤ 8	severe consciousness disturbance (GCS≤8) or rapid clinical deterioration, both demanding close brain biochemistry monitoring	effectivity of cerebral microdialysis in predicting the patient clinical outcomes	Glucose, Lactate, Pyruvate, and Glycerol	Perilesional (intact paranchyma that is ipsilateral to the damage)	GOS at 6 months	CMD-glucose GOS≥4: 1.13 (0.78–2.10) mmol/L GOS<4: 0.98 (0.85–1.39) mmol/L p = 0.520, ns CMD lactate/pyruvate ratio GOS≥4: 24.43 (18.79–27.96) GOS<4: 35.78 (33.53–38.57) p < 0.001 CMD-glycerol GOS≥4: 39.31 (33.53–53.17) mmol/L GOS<4: 64.42 (43.00–119.77) mmol/L p = 0.023
Koura et al. (1998)	Relationship between excitatory amino acid release and outcome after severe human head injury	Journal Article	United States	prospective Observational Cohort	83	Severe TBI: GCS ≤ 8	-	To test the hypothesis that the magnitude of this "Excitotoxic Surge" plays a significant role in determining 6-month patient outcome.	Glutamate	Intraparanchymally	GOS at 6 months	CMD-glutamate Threshold: >20 μmol/L GOS 0–1: 85% had mean glutamate <20 μmol/L (n = 29) GOS 2–4: 72% had mean glutamate >20 μmol/L (n = 18) CMD-glutamate vs 6-month outcome Correlation: r = 0.332, p = 0.0234
Li et al. (2008)	Extracellular glycerol in patients with severe traumatic brain injury	Journal Article	China	Retrospective Study	53	Severe TBI I (GCS range 3-7)	Severe TBI admitted to the Neuroscience Intensive Care Unit in Huanhu Hospital of Tianjin in China within 24 h.	Association between perilesional glycerol levels, cerebral perfusion pressure, cerebral blood flow and clinical outcomes	Glycerol	Peri-lesional, relatively normal tissue	Clinical outcome divided into 3 categories: Group A: The good recovery Group. B: The severe or moderate disability group; Group C: The persistent vegetative state or death group.	CMD-glycerol Good recovery (Group A, n = 26): 76.59 ± 13.03 μmol/L Severe/moderate disability (Group B, n = 11): 132.07 ± 24.53 μmol/L Persistent vegetative state or death (Group C, n = 16): 206.68 ± 25.58 μmol/L p < 0.05
Low et al. (2009)	Prediction of Outcome Utilizing Both Physiological and Biochemical Parameters in Severe Head Injury	Journal Article	Singapore	Prospective Observational Cohort	26	Severe TBI: GCS score ≤ 8	Severe TBI (GCS score of 8 or less) who were admitted to the neurocritical care unit of a tertiary hospital over a 1-year period.	evaluation of the predictibility of statistical models utilizing a combination of both physiological and biochemical variables obtained from multimodal monitoring in the neurocritical care setting.	glucose, lactate, pyruvate, and glutamate	Lesional Tissue	GOS dichotomized	Inclusion of microdialysis data significantly improved the accuracy of DT modeling for outcome prediction. - Predictive accuracy was slightly enhanced with the addition of PbtO_2_ but improved substantially when microdialysis markers were included.
Marini et al. (2022)	Correlation of brain flow variables and metabolic crisis: a prospective study in patients with severe traumatic brain injury	Journal Article	United States, Italy	Retrospective analysis of prospectively collected data	20	Severe TBI: GCS score < 8	An admission GCS score less than 8 following initial emergency room resuscitation	identification of factors contributing to development of metabolic crisis in the setting of a Multi-modality Monitoring and Goal-Directed Therapy (MM&GDTP) approach to patients	glucose, lactate, pyruvate, glycerol, and glutamate	Bifrontal, juxtaposition to PbtO2 probe	Mortality and survival GOS	CMD-LPR>40 (severe threshold) Survivors: 98/667 (14.7%) Non-survivors: 104/316 (32.9%) p < 0.05 Severe metabolic crisis duration (LPR>40, pyruvate<120, glucose<10 mmol/L) Survivors: 0.6 ± 1.0 h/patient Non-survivors: 5.8 ± 2.2 h/patient p < 0.05 Metabolic crisis events (survivors only) vs GOS Correlation: r^2^ = 0.292 (no p given)
Mazzeo et al. (2006)	Quantitation of Ischemic Events After Severe Traumatic Brain Injury in Humans: A Simple Scoring System	Journal Article	United States, Italy	Retrospective Study	172	Severe TBI: GCS score ≤ 8	-severe TBI patients (GCS 8 or less) -admitted to the Neurointensive care unit, at Medical College of Virginia, over 1993 to 2004. -patients with an already recorded ischemic score.	Association between ischemic score and clinical outcomes	glucose, lactate, pyruvate, and glutamate	Non-lesional brain tissue, right frontal lobe	GOS at 3 and 6 months	CMD-lactate vs 3-month GOS Correlation: r = −0.16, p < 0.01 CMD-lactate/glucose ratio vs 3-month GOS Correlation: r = −0.12, p < 0.01
Nelson et al. (2011)	Analyses of cerebral microdialysis in patients with traumatic brain injury: relations to intracranial pressure, cerebral perfusion pressure and catheter placement	Journal Article	Sweden	Retrospective Study	90	Severe TBI: GCS score ≤ 8	-admitted to the adult (≥15 years of age) NICU with TBI requiring mechanical ventilation (generally GCS ≤ 8), during a 5- year period, with functioning MD catheters, ICP monitoring and arterial catheters	to investigate the relations between MD parameters and ICP and/or CPP in patients with TBI	Glucose, Glycerol, Lactate and Pyruvate	Lesional Tissue (conjunction with evacuation of mass lesions)	GOS	No significant differences were observed in microdialysis parameters, including LPR or glucose, between outcome groups, indicating limited prognostic association in this cohort.
Nordström et al. (2016)	Biochemical indications of cerebral ischemia and mitochondrial dysfunction in severe brain trauma analyzed with regard to type of lesion	Journal Article	Denmark, Sweden	Prospective observational Cohort	213	Severe TBI: post-resuscitation GCS <8 (GCS motor score <5)	patients with severe TBI monitored with microdialysis during the period of November 1995 to August 20	Clinical efficacy of cerebral microdialysis	glucose, glutamate, glycerol, lactate, and pyruvate	Catheter 1: perilesional tissue Catheter B: ipsilateral or contralateral to catheter 1	Mortality in 6 months	EDH (LP ratio, glutamate, glycerol: normal/near-normal): mortality 13% overall (ischemia 100%, n = 1; mitochondrial dysfunction 0%; normal LP 8%) NML (LP ratio, glutamate, glycerol: normal/near-normal): mortality 9% overall (ischemia 33%; mitochondrial dysfunction 7%; normal LP 6%) SDH (LP ratio, glutamate, glycerol: markedly elevated, with a secondary rise at 24–60h): mortality 29% overall, highest of the four (ischemia 73%; mitochondrial dysfunction 40%; normal LP 14%) CHC (LP ratio, glutamate, glycerol: markedly elevated, no secondary rise, slow decline toward normal): mortality 14% overall (ischemia 13%; mitochondrial dysfunction 5%; normal LP 20%).
Oddo et al. (2008)	Impact of tight glycemic control on cerebral glucose metabolism after severe brain injury: A microdialysis study	Journal Article	United States	Retrospective analysis of a prospective observational cohort.	20	Severe TBI: GCS ≤8	GCS was ≤8 or they later deteriorated to this level.	To analyze the effect of tight glycemic control with the use of intensive insulin therapy on cerebral glucose metabolism in patients with severe brain injury.	Glucose	Perilesional tissue or frontal lobe	Outcome was dichotomized as survival or death at hospital discharge.	CMD-glucose, Survivors: 1.04 ± 0.56 mmol/L Non-survivors: 0.46 ± 0.23 mmol/L, brain energy crisis (glucose <0.7 mmol/L with LP ratio >40) Mortality: OR 7.36 (95% CI 1.37–39.51) p 0.02
Olivecrona et al. (2009)	Prostacyclin treatment in severe traumatic brain injury: a microdialysis and outcome study	Journal Article	Sweden	Double blinded RCT	48	Severe TBI: GCS ≤ 8	verified sTBI, GCS at intubation and sedation of 8, age 15–70 years, a first-recorded CPP of 10 mm Hg, and arrival within 24 h of trauma	effect of prostacyclin (PGI2) on brain metabolism in sTBI patients, specifically measuring the (L/P and brain glucose levels	Lactate, Pyruvate, and Glucose	Lesional tissue	GOS at 3 months	Initial CMD-L/P vs GOS (4-5 vs 1-3): n.s. Initial L/P, survivors vs deceased: n.s. (two-sided); p < 0.05 one-sided
Parafarou et al. (2011)	Cerebral perfusion pressure, microdialysis biochemistry and clinical outcome in patients with traumatic brain injury	Journal Article	Greece	Prospective observational Cohort	34	Severe TBI: GCS ≤ 8	suffered a traumatic brain injury and hospitalization in an intensive care unit was necessary (GCS ≤ 8)	relationship between CPP, ICP and microdialysis parameters and clinical outcome	Glucose, Glycerol, Pyruvate, Lactate	Right frontal lobe in diffuse injury Ipsilateral frontal lobe in case of regional damage	GOS at 6 months	Thresholds: LP ratio >37 or glycerol >72 mmol/l CMD-LP ratio: GOS ≤3: 31.86 (median 34.17, SD 8.33); GOS >3 : 24.10 (median 24.03, SD 6.88). p = 0.007 CMD-Glycerol: GOS ≤3: 80.68 μmol/l (median 66.10, SD 50.46); GOS >3: 38.64 μmol/l (median 33.18, SD 13.19). p < 0.001
Peerdeman et al. (2003)	Changes in cerebral interstitial glycerol concentration in head-injured patients; correlation with secondary events	Journal Article	The Netherlands	Prospective Observational Cohort	15	Severe TBI: GCS score ≤ 8	patients with severe traumatic brain injury and a GCS of 8 or less, admitted to the ICU of the VU University Medical Center	association between the occurrence of secondary events, the microdiasylate interstitial glycerol levels, and clinical outcome	Glucose, Glycerol, Pyruvate, Lactate	Non-lesional brain tissue	GOS at 6 months	CMD-glycerol AUC (first 24h): GOS 4-5: 991 μmol h, GOS 1-3: 4144 μmol h, p = 0.044 CMD - Peak glycerol >150 μmol/L, GOS 1-3: PPV 100%, sensitivity 60%, specificity 100%
Rajagopalan et al. (2022)	Hierarchical Cluster Analysis Identifies Distinct Physiological States After Acute Brain Injury	Journal Article	United States	Retrospective Cohort Study	32	severe TBI or acute subarachnoid hemorrhage: GCS score < 8	Patients with a GCS score < 8 were eligible for the original study if they had either severe TBI or acute SAH identified aneurysm at digital subtraction angiography and 'met institutional criteria for invasive multimodality neuromonitoring	testing the hypothesis that data-driven approaches can identify distinct physiological states from intracranial multimodality monitoring data	Glucose, Lactate, and Pyruvate	Non-lesional brain tissue	Dichotomized clinical outcome: favorable outcome: discharged to home or acute rehab unfavorable outcome: in-hospital death or discharged to chronic nursing facility	Cluster 1 (ischemia: ↑HR, ↓MAP, ↓PbtO2, ↑LPR, ↓glucose): more common in unfavorable outcome (12.8% vs 2.7%) Cluster 4 (hyperglycolysis: ↑MAP, ↑lactate, ↑pyruvate, normal LPR): more common in unfavorable (30.7% vs 14.6%) Cluster 3 (normal: ↓ICP, ↑PbtO2, ↑glucose): more common in favorable (37.1% vs 12.7%) Overall cluster distribution vs outcome: p < 0.0001
Reinert et al. (2007)	Online correlation of spontaneous arterial and intracranial pressure fluctuations in patients with diffuse severe head injury	Journal Article	Switzerland	prospective Observational Cohort	20	Severe TBI; GCS ≤9	suffering from diffuse severe head injury, with a GCS ≤ 9 at referral time, were included into the study.	online analysis technique to monitor the correlation (AI*ρ*) between the spontaneous fluctuations of the MABP and ICP	Glucose, Lactate	Frontal lobe	GOS at 12 months	CMD-glucose GOS 4-5: 2045 ± 196 μmol/l, GOS 1-3: 1273 ± 49 μmol/l, p < 0.0001 CMD-lactate GOS 4-5: 2437 ± 110 μmol GOS 1-3: 1590 ± 120 μmol/l, p < 0.0001
Sanchez et al. (2013)	Neuromonitoring with microdialysis in severe traumatic brain injury patients	Journal Article	United States	prospective Observational Cohort	12	TBI: Group 1: GCS 3-6 Group 2: GCS 7-9	not provided	Hypothesis: Neuromonitoring with microdialysis has the potential for early detection of metabolic derangements associated with TB	Glucose, Lactate, and Pyruvate	NA	GOS at the time of discharge	Glucose, high (>2.6 mmol/L) GOS 1–3: p < 0.0001 Lactate high (≥8.9 mM) in GOS 1–3: p < 0.001 LPR, high (>25) in GOS 1–3: p 0.01 Pyruvate, low (<120 μM) in GOS 1–3: p < 0.001 lactate/glucose ratio, higher in GOS 4–5: p < 0.0001
Sánchez-Porras et al. (2012)	Long' pressure reactivity index (L-PRx) as a measure of autoregulation correlates with outcome in traumatic brain injury patients	Journal Article	Germany, United Kingdom	Retrospective Study	29	Severe TBI: GCS ≤ 8	with a GCS score of≤8 on presentation, or within 48 h of trauma	Correlation between low frequency changes in ICP and CPP with clinical outcome at 6 months	glucose, glutamate, lactate, and pyruvate	NA	GOS at 6 months	. High CMD-glucose, CMD-lactate level (p < 0.001), and CMD-LPR (p < 0.01) was observed in patients with GOS 1–3. Pyruvate level was low in patients with GOS 1–3 (p < 0.001). LGR was higher in patient with better outcome (GOS 4–5) CMD - Glutamate vs GOS: r = −0.442, p = 0.016 CMD- Lactate vs GOS: r = −0.426, p = 0.021 CMD- lactate Non-survivors: 5.8 ± 1.8; mM Survivors: 3.6 ± 1.5 mM, p = 0.033; CMD LP ratio Non-survivors: 72.9 ± 54.6 survivors 31.3 ± 8.8, p = 0.024
Stein et al. (2012)	Early cerebral metabolic crisis after TBI influences outcome despite adequate hemodynamic resuscitation	Journal Article	United States	Prospective Observational Cohort	89	Moderate to severe TBI: a GCS of ≤8 or a GCS of 9–14 with computerized tomography brain scans demonstrating intracranial bleeding	GCS of ≤ 8 or an admission GCS of 9–12 with computerized tomography brain scans demonstrating intracranial bleeding	correlation between frequency of metabolic crisis and patient clinical outcome	glucose, glutamate, glycerol, lactate, and pyruvate	Non-lesional brain tissue	GOSE at 6 months	Metabolic crisis duration (h), GOSE ≥7: 1.4 GOSE ≤6: 25.2, p = 0.011 CMD – LP ratio GOSE ≥7: 25.4 GOSE ≤6: 32.8, p = 0.011 CMD-glucose <0.8 mmol/L (% time), GOSE ≥7: 9.1% 47.3%, p = 0.021 CMD-glutamate, GOSE ≥7: 3.8 μmol/L GOSE ≤6: 8.8 μmol/L, p = 0.027 Glucose, Lactate, pyruvate, glycerol: n.s.
Thelin et al. (2014)	Microdialysis Monitoring of CSF Parameters in Severe Traumatic Brain Injury Patients: A Novel Approach	Journal Article	Sweden	Prospective Observational Cohort	14	Severe TBI: (GCS 3–8 at admission)	Suffering from severe TBI were included between January 1, 2010 and December 31, 2012	Correlation between CSF microdialysis, regional brain microdialysis and clinical outcome	Lactate, Pyruvate, Glycerol, and Glucose	Perilesional (adjacent to ventricular drainage in effected hemisphere)	GOSE at 6 months	No significant difference in outcome was found using the LPR, or any of the regional CMD-Brain monitoring in analyzed cohort.
Vespa et al. (2003)	Persistently Low Extracellular Glucose Correlates With Poor Outcome 6 Months After Human Traumatic Brain Injury Despite a Lack of Increased Lactate: A Microdialysis Study	Journal Article	United States	Prospective Observational Cohort	30	Severe TBI: GCS of 8 or less or with evidence of traumatic mass lesion on a computerized tomographic scan and GCS score of 12 or less	TBI patients with - GCS of 8 or less Or -with evidence of traumatic mass lesion on a computerized to- mographic scan and GCS score of 12 or less	correlation between microdiasylate Glucose levels and patient clinical outcome	glucose, glutamate, lactate, and pyruvate	Dorsolateral frontal white matter (ipsilateral to ventricle)	GOSE at 6 months	CMD-glucose vs outcome hour-based trend: p = 0.0001; case-based: n.s. (p = 0.79) CMD-LGR hour-based trend: p = 0.0001; case-based (all): p = 0.01; case-based (initial 50h): p = 0.04 CMD-lactate vs outcome: n.s. in case-based analysis Prolonged low glucose (>20% of time) worse 6-month GOSe: OR 2.6 (95% CI 1.96–3.6), p < 0.001 Prolonged low glucose → worse Disability Rating Scale: OR 3.25 (95% CI 0.85–12.5), p < 0.001
Vespa et al. (2006)	Intensive insulin therapy reduces microdialysis glucose values without altering glucose utilization or improving the lactate/pyruvate ratio after traumatic brain injury*	Journal Article	United States	Non-randomized clinical trial with retrospective data analysis	47	Moderate to severe TBI: - GCS ≤ 8 or evidence of traumatic mass lesion on computed tomographic scan and GCS ≤12	TBI patients with GCS of 8 or less Or -with evidence of traumatic mass lesion on a computerized to- mographic scan and GCS of 12 or less	association between intensive insulin therapy, cerebral microdiasylates and clinical outcomes	glucose, glutamate, lactate, and pyruvate	Non-lesional tissue	GOSE at 6 months	% time CMD-glucose <0.2 mmol/L: mortality: R^2^ = 0.39, p < 0.04
Zauner et al. (1997)	Continuous Monitoring of Cerebral Substrate Delivery and Clearance: Initial Experience in 24 Patients with Severe Acute Brain Injuries	Journal Article	United States	prospective Observational Cohort	24	Severe TBI; GCS ≤8	Patients who were older than 16 years, suffered severe head injury, and sustained an admission GCS of 8 or less were studied. Only patients who underwent ventriculostomy-ICP monitoring and/or underwent cranial surgery were selected.	brain tissue oxygen tension was used to differentiate patients at risk for brain ischemia and to predict outcome.	Glucose, Lactate	Frontal cortex or white matter (usually noncontused area)	GOS at 3 or 6 months after injury	CMD-glucose: Group I good outcome: 986 μmol/L, Group II moderate/severe disability:89 μmol/L, Group III death/vegetative: 639 μmol/L, p < 0.05 (Group III lower) CMD - lactate: Group I:1031 μmol/L, Group II 1180 μmol/L, Group III 1642 μmol/L, p < 0.05 (Group III higher)

Table 1 summarizes study characteristics including study design, sample size, monitored CMD analytes probe placement, reported functional outcome measures, results and conclusions.

*Abbreviations*: CBF, cerebral blood flow; CMD, cerebral microdialysis; CPP, cerebral perfusion pressure; DC, decompressive craniotomy; DTI, delayed traumatic injury; GCS, Glasgow Coma Scale; GOS, Glasgow Outcome Scale; GOSE, Glasgow Outcome Scale–Extended; ICP, intracranial pressure; ICU, Intensive Care Unit; L/G, lactate glucose; LGR, lactate glucose ratio; L/P, lactate pyruvate ratio; LPR, lactate-to-pyruvate ratio; MABP, mean arterial blood pressure; MC, metabolic crisis; ns, not significant; PbtO2, brain tissue oxygen tension; PCO_2_, partial pressure of carbon dioxide; sTBI, severe traumatic brain injury; TBI, traumatic brain injury.

### 3.2 Risk of bias assessment

Details on risk of bias assessments are presented in Appendix B. Among all the included studies, nineteen were judged to have a high risk of bias, ten a moderate risk, and 4 low risk of bias, indicating substantial methodological limitations across the available evidence.

### 3.3 CMD methods

CMD-lactate and CMD-glucose were the most frequently studied analytes (each reported in 26/33 studies, 79%), whereas glycerol was evaluated in 12/33 studies (36%). CMD monitoring was usually incorporated into a multimodal neuromonitoring strategy alongside ICP measurements. In most studies, CMD probes were placed in injured or perilesional brain tissue, often in the subcortical white matter near the primary lesion (Paraforou et al., 2011; Gopinath et al., 2000; Hägglund et al., 2022; Hejčl et al., 2011; Ho et al., 2008; Karathanou et al., 2011; Li et al., 2008; Nordström et al., 2016; Oddo et al., 2008; Olivecrona et al., 2009; Thelin et al., 2014; Gupta et al., 2017). In four studies, catheters were positioned on the side of maximal injury or swelling as defined by CT imaging (Hlatky et al., 2007; Igarashi et al., 2017; Low et al., 2009; Nelson et al., 2011). Two studies did not mention probe location at all (Sanchez et al., 2013; Sánchez-Porras et al., 2012), and four described the anatomical position without specifying whether it was in injured or non-injured tissue (Koura et al., 1998; Marini et al., 2022; Reinert et al., 2007; Vespa et al., 2003).

Across studies, probe placement strategies were generally aimed at sampling lesional or perilesional tissue; however, probe location was rarely analyzed as an independent determinant of CMD values or outcome. Only one study (Nelson et al., 2011) explicitly compared CMD markers from perilesional versus non-lesional regions and found no significant association between probe location and clinical outcome, while another reported outcome associations for penumbral probes but did not vary or model probe location as a prognostic factor because position was standardized across patients (Hejčl et al., 2011).

Monitoring duration also varied widely. Fourteen studies monitored patients for approximately 3–5 days following injury (Clausen et al., 2005a, 2005b; Eiden et al., 2019; Hägglund et al., 2022; Igarashi et al., 2017; Koura et al., 1998; Li et al., 2008; Marini et al., 2022; Mazzeo et al., 2006; Oddo et al., 2008; Olivecrona et al., 2009; Sánchez-Porras et al., 2012; Vespa et al., 2006; Gupta et al., 2017), and nine extended monitoring up to about 7–10 days (Paraforou et al., 2011; Ho et al., 2008; Igarashi et al., 2017; Karathanou et al., 2011; Rajagopalan et al., 2022; Stein et al., 2012; Thelin et al., 2014; Vespa et al., 2003; Zauner et al., 1997). Two studies (Igarashi et al., 2017; Peerdeman et al., 2003) focused exclusively on the results of the first 24 h post-injury, whereas one study (Hejčl et al., 2011) reported data collected for up to 19 days, and five did not specify monitoring duration (Goodman et al., 1999; Hlatky et al., 2007; Low et al., 2009; Reinert et al., 2007; Sanchez et al., 2013). Such variability in monitoring windows likely contributed to differences in observed metabolic dynamics and CMD - outcome associations.

Catheter characteristics and perfusion rates were similarly heterogeneous. Most studies (n = 19) used a 20-kDa microdialysis catheter; one study used both 20- and 100-kDa catheters (Nordström et al., 2016), and seven studies (Paraforou et al., 2011; Hägglund et al., 2022; Oddo et al., 2008; Olivecrona et al., 2009; Peerdeman et al., 2003; Stein et al., 2012; Gupta et al., 2017) reported using CMA70 catheters, while one study used both CMA70 and CMA100 devices (Goodman et al., 1999). Five studies (Gopinath et al., 2000; Hejčl et al., 2011; Marini et al., 2022; Rajagopalan et al., 2022; Sanchez et al., 2013) did not report the molecular-weight cutoff. Perfusion rates most commonly were 0.3 μL/min (n = 17), while 2 μL/min was the next most frequent rate. Six studies (Paraforou et al., 2011; Hejčl et al., 2011; Karathanou et al., 2011; Koura et al., 1998; Rajagopalan et al., 2022; Sanchez et al., 2013) did not report perfusion speed, and three (Chamoun et al., 2010; Reinert et al., 2007; Vespa et al., 2006) used alternative flow rates of 3, 0.5, and 1 μL/min. Overall, these differences in probe placement, monitoring duration, catheter properties, and perfusion settings underscore substantial methodological heterogeneity across CMD studies.

### 3.4 Outcome measures

Functional outcome was most frequently assessed using the GOS at 6 months (13/33, 39%) (Paraforou et al., 2011; Chamoun et al., 2010; Eiden et al., 2019; Gopinath et al., 2000; Hejčl et al., 2011; Ho et al., 2008; Karathanou et al., 2011; Koura et al., 1998; Mazzeo et al., 2006; Peerdeman et al., 2003; Sánchez-Porras et al., 2012; Zauner et al., 1997; Gupta et al., 2017), with additional assessments at 3 months (Clausen et al., 2005a, 2005b; Mazzeo et al., 2006; Olivecrona et al., 2009; Zauner et al., 1997) or hospital discharge in several cohorts (Igarashi et al., 2017; Marini et al., 2022; Nelson et al., 2011; Rajagopalan et al., 2022; Sanchez et al., 2013) GOSE at 6 months was reported in 6 studies (Hägglund et al., 2022; Hlatky et al., 2007; Stein et al., 2012; Thelin et al., 2014; Vespa et al., 2003, 2006), while 5 studies reported mortality as the sole outcome (Chamoun et al., 2010; Goodman et al., 1999; Igarashi et al., 2017; Marini et al., 2022; Oddo et al., 2008). Dichotomization of GOSE varied, with favorable outcome defined as GOSE 5–8 (Vespa et al., 2003, 2006), 6–8 (Thelin et al., 2014), or ≥7 (Stein et al., 2012) across different studies.

### 3.5 Brain metabolism and clinical outcomes

#### 3.5.1 Glucose

CMD-glucose was reported in 26 studies, of which 7 evaluated its association with outcome. Studies where glucose was evaluated only as a component of a composite metabolic crisis or multimodal cluster are reported in Section C.5.6 and C.5.7. Five studies found that higher CMD-glucose concentrations were associated with better outcome (Igarashi et al., 2017; Oddo et al., 2008; Reinert et al., 2007; Vespa et al., 2003; Zauner et al., 1997), defined as survival or higher GOS/GOSE scores. One study reported a negative association between CMD-glucose and survival (Sanchez et al., 2013). One study (Goodman et al., 1999) found no significant association between CMD-glucose levels and patient outcomes when the probe was placed in non-lesional brain tissue. Notably, two studies found that the duration of exposure to critically low glucose, rather than a single glucose value, was the more meaningful predictor of outcome: Vespa et al. (2003) reported that prolonged low glucose exposure was independently associated with poor 6-month GOSE and Stein et al. (2012) similarly demonstrated that the percentage of monitoring time spent below the 0.8 mmol/L threshold was significantly greater in patients with unfavorable outcome.

In addition, three studies evaluated the lactate/glucose (L/G) ratio as an indicator of enhanced glycolysis and found that higher L/G ratios were associated with increased mortality or unfavorable outcome, with non-survivors typically exhibiting L/G ratios between approximately 4.5 and 6.7 (Goodman et al., 1999; Igarashi et al., 2017; Mazzeo et al., 2006).

Probe placement varied across studies. Two studies (Vespa et al., 2003) (Zauner et al., 1997) used non-lesional placement yet found significant glucose - outcome associations, indicating that non-lesional probes can detect clinically meaningful metabolic changes. By contrast, Goodman et al. (1999), who also used non-lesional placement, found no significant glucose association. Two studies (Igarashi et al., 2017; Oddo et al., 2008) placed the probe perilesionally and found a significant glucose - outcome association.

Across these studies, inconsistent probe locations (lesional versus non-lesional), different monitoring windows, and variable outcome time points likely contributed to these divergent findings, and clinically meaningful glucose or L/G thresholds were rarely prespecified. Comparability and identification of clinically robust cut-off values is therefore limited.

#### 3.5.2 Lactate and pyruvate

CMD-lactate was reported in 26 studies, and 9 of these described a statistically significant association between lactate concentrations and outcome (Eiden et al., 2019; Goodman et al., 1999; Hlatky et al., 2007; Ho et al., 2008; Igarashi et al., 2017; Mazzeo et al., 2006; Reinert et al., 2007; Sanchez et al., 2013; Sánchez-Porras et al., 2012). In patients with poor outcome, reported lactate values ranged from approximately 0.8 to 7.0 mmol/L. Individuals with more favorable outcomes tended to have lower lactate levels. Pyruvate was evaluated in 22 studies, and 3 studies reported significant associations between low CMD-pyruvate concentrations and unfavorable clinical outcome (Eiden et al., 2019; Hlatky et al., 2007; Sanchez et al., 2013). In these cohorts, pyruvate levels below approximately 50 μmol/L were more frequently observed in patients who died or had poor functional recovery.

Among the nine studies reporting significant lactate–outcome associations, probe placement varied considerably. Studies with confirmed perilesional or injured tissue placement (Ho et al., 2008) and those targeting the site of maximal injury or swelling (Hlatky et al., 2007; Igarashi et al., 2017) contributed the majority of significant findings. Two studies reporting significant lactate associations did not describe probe location at all (Sanchez et al., 2013; Sánchez-Porras et al., 2012), which limits interpretation of their absolute lactate values. Notably, Goodman et al. (1999) used a non-lesional probe placement yet still found a significant lactate-outcome association, suggesting that pathological lactate elevation may extend beyond the perilesional zone. For the three studies reporting significant pyruvate associations, probe location was specified in only one, which was placed at the site of maximal injury (Hlatky et al., 2007); the remaining studies provided no location information. The heterogeneity in probe placement across lactate and pyruvate studies, combined with the absence of location data in two, limits comparison of absolute concentrations and contributes to the wide variability in reported threshold values.

Absolute metabolite concentrations also varied widely across cohorts, likely reflecting differences in probe location, sampling intervals, and analytical methods. Although elevated lactate levels were generally associated with mortality or unfavorable outcome, methodological heterogeneity and the absence of standardized thresholds precluded consistent prognostic cut-offs. Similar variability in probe placement and sampling strategies for pyruvate further limited comparability across studies and weakened inference regarding its independent prognostic value.

#### 3.5.3 Lactate/pyruvate (L/P) ratio

The L/P ratio was reported in 22 studies, with most analysis focusing on its role as a marker of impaired oxidative metabolism. Across studies, elevated L/P ratios were generally associated with higher mortality and lower GOS or GOSE scores. Survivors tended to maintain lower ratios, often below approximately 25-40. Only a minority of studies defined explicit L/P thresholds for metabolic crisis, and these cut-offs varied, with values between 25 and 37 most frequently used.

Studies reporting L/P ratio data predominantly placed probes in perilesional or injured subcortical white matter (Paraforou et al., 2011) (Gopinath et al., 2000; Hägglund et al., 2022; Hejčl et al., 2011; Ho et al., 2008; Karathanou et al., 2011; Li et al., 2008; Nordström et al., 2016; Oddo et al., 2008; Olivecrona et al., 2009; Thelin et al., 2014; Gupta et al., 2017), or at the site of maximal injury as defined by CT imaging (Hlatky et al., 2007; Igarashi et al., 2017; Low et al., 2009; Nelson et al., 2011). Four studies described only the anatomical catheter position without specifying whether tissue was injured or non-injured (Koura et al., 1998; Marini et al., 2022; Reinert et al., 2007; Vespa et al., 2003), and two studies didn't mention probe location entirely (Sanchez et al., 2013; Sánchez-Porras et al., 2012). Whether probe placement modifies the prognostic value of the L/P ratio, and whether the ratio retains discriminatory value in non-lesional tissue, cannot be determined from the available data as no included study directly compared L/P thresholds between lesional and non-lesional monitoring sites.

Furthermore, most studies analyzed L/P as a continuous variable or compared survivors and non-survivors without prespecified cut-offs, which, combined with differences in probe location and monitoring windows, precluded the establishment of a uniform prognostic threshold. Taken together, these findings support a directional association between higher L/P ratio and worse outcome but do not yield a standardized L/P criterion suitable for clinical prognostication.

#### 3.5.4 Glutamate

CMD-glutamate was measured in 16 studies, and 6 of these reported a significant association between elevated CMD-glutamate concentrations and mortality or unfavorable functional outcome (Chamoun et al., 2010; Eiden et al., 2019; Gopinath et al., 2000; Igarashi et al., 2017; Koura et al., 1998; Stein et al., 2012). Two studies defined an elevated glutamate threshold of >20 μmol/L (Chamoun et al., 2010; Koura et al., 1998), above which patients more often experienced poor outcomes, whereas others described higher median or peak glutamate levels in non-survivors without specifying formal cut-offs. Probe location again varied, with some studies sampling non-lesional tissue and others perilesional or lesional regions, and several did not clearly distinguish between these compartments. Outcome definitions and follow-up time points also differed between studies. One study identified distinct temporal glutamate patterns, reporting that normalization of initially abnormal glutamate levels over the first 120 h was associated with better 6-month outcome, whereas persistently elevated or progressively rising glutamate was linked to higher mortality (Chamoun et al., 2010).

Among the six studies reporting significant glutamate - outcome associations, probe placement varied but notably three studies used non-lesional or healthy appearing tissue (Chamoun et al., 2010; Eiden et al., 2019; Stein et al., 2012). One study used perilesional placement (Gopinath et al., 2000), one targeted the site of maximal injury (Igarashi et al., 2017), and one described the anatomical position without specifying injury proximity (Koura et al., 1998). The fact that significant glutamate - outcome associations were detected in both non-lesional and perilesional tissue across these studies suggests that pathological glutamate elevation may not be confined to the immediate injury zone, and that glutamate may be detectable as a prognostic marker regardless of probe placement relative to the primary lesion.

Overall, the available data suggest that sustained or markedly elevated glutamate levels are associated with worse outcome, but heterogeneous definitions and limited adjustment for confounders limit the strength and generalizability of this association.

#### 3.5.5 Glycerol

CMD-glycerol was evaluated in 12 studies, 6 of which reported a significant association between higher CMD-glycerol levels and mortality or unfavorable functional outcome (Paraforou et al., 2011; Hejčl et al., 2011; Igarashi et al., 2017; Karathanou et al., 2011; Li et al., 2008; Peerdeman et al., 2003). Studies that reported explicit thresholds typically described poor outcome or death at peak glycerol concentrations exceeding approximately 150–200 μmol/L, whereas survivors more often had lower peak values (62 to 124 μmol/L). In one study (Clausen et al., 2005a), brain tissue glycerol concentrations declined significantly during the first four days after injury in all patients, with comparable exponential decreases in both favorable and poor outcome groups and no significant differences between them.

Among the six studies reporting significant glycerol - outcome, probe placement was predominantly perilesional or in injured subcortical white matter (Paraforou et al., 2011; Hejčl et al., 2011; Karathanou et al., 2011; Li et al., 2008), with one study targeting the site of maximal injury (Igarashi et al., 2017). The majority of significant findings therefore came from studies monitoring tissue at or adjacent to the primary lesion, suggesting that perilesional placement may yield stronger and more consistent glycerol - outcome signals. However, one study reporting a significant association used a healthy tissue probe (Peerdeman et al., 2003), indicating that elevated glycerol can be detected in non-lesional tissue under certain conditions. The one study (Clausen et al., 2005a) that reported no significant glycerol - outcome difference also used a healthy tissue probe, yielding a null result. The mixed findings from healthy tissue probes suggest that glycerol detectability in non-lesional tissue may depend on factors such as injury severity, proximity to the lesion, or timing of sampling rather than probe location alone.

However, thresholds and units were not standardized, sample sizes were small, and most studies did not adjust for core TBI prognostic variables, limiting the robustness and generalizability of these findings.

#### 3.5.6 Biochemical patterns in cerebral microdialysis

Eight studies (Eiden et al., 2019; Marini et al., 2022; Mazzeo et al., 2006; Nordström et al., 2016; Oddo et al., 2008; Rajagopalan et al., 2022; Stein et al., 2012; Gupta et al., 2017) analyzed combinations of analytes as a composite construct in relation to outcome, rather than reporting analytes individually. These took three broadly related forms: threshold-defined metabolic crisis or brain energy crisis combining low CMD-glucose with an elevated LPR (Marini et al., 2022; Oddo et al., 2008; Stein et al., 2012), pyruvate-based separation of ischemia from mitochondrial dysfunction (Marini et al., 2022; Nordström et al., 2016; Gupta et al., 2017) and data-driven metabolic states or clusters derived from multiple analytes (Eiden et al., 2019; Rajagopalan et al., 2022) alongside one composite ischemic score (Mazzeo et al., 2006)^.^ Across these studies, a greater burden of the unfavorable metabolic state, whether expressed as its presence, severity or duration, was consistently associated with higher mortality and worse functional outcome. However, the defining thresholds varied widely (LPR cut-offs of 25 to 40; glucose cut-offs of 0.7 to 0.8 mmol/L, definition of metabolic crisis).

Furthermore, across studies that evaluated CMD analytes with biochemical patterns, probe placement spanned both lesional and non-lesional tissue. Three studies used perilesional or injured subcortical white matter (Nordström et al., 2016; Oddo et al., 2008; Gupta et al., 2017), while four used healthy or normal appearing brain tissue (Eiden et al., 2019; Mazzeo et al., 2006; Rajagopalan et al., 2022; Stein et al., 2012). One study described the anatomical position without specifying injury proximity (Marini et al., 2022). The fact that unfavorable composite metabolic states were consistently associated with worse outcome regardless of whether probes were in lesional or non-lesional tissue suggests that the metabolic crisis construct may capture more diffuse pathophysiology than single-analyte thresholds alone.

#### 3.5.7 Multimodal monitoring

Three studies (Low et al., 2009; Marini et al., 2022; Rajagopalan et al., 2022) specifically examined multimodal monitoring strategies that combined CMD analytes with other physiological parameters, most commonly intracranial pressure, cerebral perfusion pressure, and brain tissue oxygenation. These studies consistently reported that multimodal models incorporating CMD data provided better discrimination of mortality or functional outcome than models based on single CMD analytes alone.

Probe placement differed across the three multimodal studies. One study positioned the catheter at the site of maximal injury or swelling as defined by CT imaging (Low et al., 2009), one described the anatomical position without specifying whether tissue was injured or non-injured (Marini et al., 2022), and one used healthy or normal appearing brain tissue (Rajagopalan et al., 2022). The heterogeneity in probe location across these three studies means that multimodal CMD signals were sampled from different tissue compartments, which may have influenced both the absolute analyte values and the relative contribution of individual CMD variables within each multimodal model. This limits direct comparison of the models and their predictor weights across studies.

Nonetheless, these multimodal studies differed in their modeling strategies, predictor sets, and outcome definitions, and none underwent external validation, so their findings should be interpreted as exploratory rather than as ready-to-use prognostic tools.

### 3.6 Certainty of evidence

The certainty of evidence for each CMD analyte is summarized in Table 2 and was rated using the GRADE approach (Guyatt et al., 2008), following prognostic factor specific guidance (GRADE Guideline 28) (Foroutan et al., 2020). Certainty was mainly limited by risk of bias in predominantly observational studies, indirectness due to substantial heterogeneity in CMD methodology (especially probe location, monitoring duration, and catheter characteristics), and imprecision arising from small sample sizes, non-comparable effect estimates, and inconsistently defined biomarker thresholds. Consequently, most analytes were graded as having low to very low certainty, with glycerol and L/P ratio showing the most consistent directional association but still being downgraded because of methodological variability and limited sample sizes. Due to heterogeneity, no quantitative meta-analysis was performed.

**Table 2:** Certainty of evidence (GRADE assessment).

CMD MEASURES	NO. OF STUDIES	STUDY DESIGN	RISK OF BIAS	INCONSISTENCY	INDIRECTNESS	IMPRECISION	PUBLICATION BIAS	CERTAINTY OF EVIDENCE
GLUCOSE	26	Observational	High	No	Yes	No	Unclear	Low - Very Low
LACTATE	26	Observational	High	No	Yes	No	Unclear	Low - Very Low
PYRUVATE	22	Observational	High	No	Yes	No	Unclear	Very Low
L/P RATIO	22	Observational	High	No	Yes	No	Unclear	Low
GLUTAMATE	16	Observational	High	Yes	Yes	No	Unclear	Very Low
GLYCEROL	12	Observational	Moderate	No	Yes	Yes	Unclear	Low
BIOCHEMICAL PATTERNS	8	Observational	High	No	Yes	Yes	Unclear	Very Low
MULTIMODAL MONITORING	3	Observational	High	No	Yes	Yes	Unclear	Very Low

Table 2 summarizes the certainty of evidence for associations between CMD biomarkers and clinical outcomes, assessed using the GRADE^1^ approach for prognostic factor research (GRADE Guideline 28). Certainty levels were rated as high, moderate, low, or very low. Indirectness was downgraded when CMD probe location (lesional, perilesional vs non-lesional vs unclear), monitoring windows and catheter characteristics varied substantially across studies. Imprecision was downgraded when evidence relied on small cohorts or used inconsistent/unstable biomarker thresholds preventing clinically meaningful risk stratification.

Abbreviations: CMD, cerebral microdialysis; L/P ratio, lactate/pyruvate ratio.

## 4 Discussion

This systematic review synthesizes current evidence on CMD biomarkers and their associations with mortality and functional outcome after moderate-to-severe TBI. Lower CMD-glucose, higher CMD-lactate, higher L/P ratio, elevated glutamate, and higher CMD-glycerol were generally associated with worse outcomes, whereas higher CMD-glucose and lower CMD-lactate were more often observed in patients with better recovery, although findings were not uniform across studies. Glycerol, despite being evaluated in fewer cohorts, showed the most consistent directional association with poor outcome, but certainty of evidence remained low because of methodological limitations.

Each CMD analyte reports on a different step in the injured brain's energy metabolism and structural integrity and the associations described above are best interpreted against this background. Glucose is the principal metabolic fuel of the brain: it is delivered from the circulation, broken down through glycolysis to pyruvate, and pyruvate then enters the tricarboxylic acid cycle, where oxidative phosphorylation regenerates ATP (Stovell et al., 2023). The glucose concentration measured in the interstitium is therefore what remains once delivery and consumption have balanced, and a low CMD glucose signals neuroglycopenia, which may result from inadequate supply, as in systemic hypoglycemia or reduced cerebral blood flow, or from excessive demand, as in hyperglycolysis, seizures, or cortical spreading depolarization (Stovell et al., 2023; Hutchinson et al., 2015). When oxygen delivery or mitochondrial function becomes insufficient, pyruvate is diverted to lactate, and the L/P ratio rises accordingly. This ratio reflects the redox state and is the most established CMD marker of cerebral energy metabolism, with values of 25 or above, or 40 in some studies, regarded as pathological, and values above 40 accompanied by low interstitial glucose defining a metabolic crisis (Stovell et al., 2023; Hutchinson et al., 2015). Elevated interstitial glutamate reflects excitotoxic injury, in which excessive excitatory neurotransmitter release and impaired re-uptake drive calcium influx and downstream neuronal damage (Chamoun et al., 2010). Glycerol is a structural component of cell membranes and its appearance in the interstitium signals phospholipid breakdown and loss of membrane integrity, providing a marker of cellular degradation rather than of energy state alone (Hillered et al., 2005). Framed this way, the analytes are not interchangeable outcome correlates but complementary readouts of distinct pathophysiological processes, which is central to interpreting both their prognostic signal and their heterogeneity across studies.

Several studies indicated that CMD findings were most informative when interpreted alongside other neuromonitoring modalities such as ICP, CPP, PbtO_2_ and PRx. Multimodal approaches that combined CMD analytes with intracranial and oxygenation measures tended to improve discrimination of mortality and functional outcome compared with CMD alone, supporting the concept that CMD primarily adds value by offering a more comprehensive understanding of cerebral pathophysiology after TBI and within an integrated monitoring framework rather than as a stand-alone prognostic tool (Low et al., 2009; Marini et al., 2022; Rajagopalan et al., 2022). It is important to emphasize that the principal, intended clinical role of CMD was not designed to predict outcome but rather the purpose is to monitor the injured brain in real time and to guide treatment in the individual patient. At the bedside, a fall in CMD glucose or a rise in the L/P ratio may lead the clinician to optimize CPP, adjust sedation or glycemic targets, or escalate ICP-directed therapy, often before deterioration is evident clinically or on ICP (Carteron et al., 2017). The present review therefore answers a narrow question, whether isolated analyte values predict long-term outcome, and our cautious conclusions do not call into question the value of CMD as a real-time monitor. These observations are consistent with recent multimodal monitoring literature, which increasingly regards CMD as an investigational component within broader neurocritical-care strategies (Baker et al., 2025; Vitt et al., 2023; Casault et al., 2022).

Our findings broadly align with an earlier systematic review on CMD and outcomes in TBI, which also reported associations between metabolic disturbances and functional outcome but did not apply a prognostic-factor GRADE framework (Zeiler et al., 2017b). CMD has also been shown to complement other intracranial monitoring modalities in TBI (Low et al., 2009; Marini et al., 2022; Rajagopalan et al., 2022). Notably, several studies have reported associations between metabolic disturbances detected via cerebral microdialysis and elevated ICP (Chamoun et al., 2010; Eiden et al., 2019; Goodman et al., 1999; Hejčl et al., 2011; Hlatky et al., 2007; Ho et al., 2008; Koura et al., 1998; Li et al., 2008; Nelson et al., 2011; Oddo et al., 2008; Rajagopalan et al., 2022; Reinert et al., 2007). By identifying metabolic derangements, such as elevated CMD-lactate and low CMD-glucose, CMD may provide insights into the metabolic patterns associated with poorer functional outcomes after TBI in adjunct to known prognosticators (Sanchez et al., 2013; Guilfoyle et al., 2021).

By focusing specifically on CMD analytes as prognostic factors and rating the certainty of evidence, we show that, despite repeated directional associations, the overall certainty for individual analytes remains low to very low. The main reasons are the predominance of small observational cohorts, substantial heterogeneity in probe placement (lesional vs perilesional vs non-lesional tissue), variable monitoring windows and catheter characteristics, and inconsistent outcome measures and dichotomization schemes (Nelson et al., 2011). Together, these factors limit comparability across studies and preclude definition of clinically robust thresholds for prognostic stratification.

In addition, CMD was used in different ways across studies, ranging from purely diagnostic monitoring to components of ICP-guided treatment protocols, which complicates assessment of its prognostic utility. Outcome assessments varied in scale (GOS vs GOSE vs mortality) and timing (3, 6, or 12 months), and reporting units and thresholds, particularly for glycerol, were often non-standardized or absent. Beyond methodological and reporting issues, CMD implementation also entails substantial costs related to catheters, analyzers and staff time, which must be weighed against the currently limited and uncertain evidence of patient-centered benefit (Zimphango et al., 2022). In an era of rising healthcare costs and increasing pressure on resource allocation, establishing prognostic utility through standardized studies is a necessary prerequisite before broader questions of cost-effectiveness and routine clinical implementation can be meaningfully addressed.

From a bedside perspective, these findings carry a measured but useful message. The directional consistency observed for glycerol and the L/P ratio suggests that these analytes may reasonably raise or lower a clinician's index of suspicion for evolving secondary injury when they are interpreted in context and alongside concurrent ICP, CPP, PRx and PbtO2 data, rather than in isolation. What the current evidence does not support is the use of any single analyte value or any single published threshold, as an independent determinant of long-term prognosis or as a trigger for prognostic counseling. In other words, CMD can meaningfully inform the moment-to-moment physiological picture, but it cannot yet be treated as a validated standalone predictor of patient's clinical outcome.

Despite our literature search extending to 2026, no eligible studies were identified after 2022. Several converging factors may account for this gap. CMD research requires specialized invasive equipment and expertise, which constrains the pace at which new prospective cohorts can be completed and published. Concurrently, the field has progressively shifted its focus toward integrated multimodal neuromonitoring frameworks, potentially redirecting investigative effort away from single-analyte prognostic studies. Looking ahead, several ongoing multimodal monitoring trials incorporate cerebral microdialysis as a secondary or exploratory endpoint and may generate prognostically informative CMD data in the coming years.

Building on these limitations, we propose the outline of an ideal study designed specifically to establish the prognostic value of CMD. Such a study would enroll a well-defined cohort of adults with moderate to severe TBI (GCS 3 to 12), thereby minimizing selection bias. Catheter placement would be standardized and reported relative to focal lesions using imaging, with a stratification of perilesional versus radiologically normal tissue, since probe location is a principal source of between-study heterogeneity. Monitoring would follow a fixed protocol with continuous sampling across a defined early window (for example, the first five to seven days after injury), and analytes, units and thresholds, would be prespecified rather than derived post hoc. CMD data would be collected within a full multimodal framework alongside ICP, CPP, PRx and PbtO2, both to reflect real clinical use and to allow the prognostic contribution of CMD on established monitors. Functional outcome would be assessed with ideally the GOSE, at prespecified time points of three, six to twelve months. Finally, analysis would be adjusted for validated TBI prognostic factors, including age, admission GCS, pupillary reactivity and CT characteristics (for example, Marshall or Rotterdam scores), consistent with established prognostic modeling frameworks. Consistent design and reporting across future studies would allow their findings to be compared and combined, which is what ultimately determines whether any of these analytes hold genuine prognostic value.

Overall, although CMD biomarkers frequently correlate with cerebral metabolic disturbances and reported outcomes, the present evidence base is too heterogeneous and of too low certainty to support clinically actionable prognostic stratification. Existing CMD studies are not yet designed in a sufficiently standardized manner to answer key questions about prognostic accuracy or the (cost-)effectiveness of CMD-guided therapy, underscoring the need for high-quality, prognostically oriented cohort studies before interventional trials can be meaningfully pursued.

## 5 Conclusion

Current CMD research documents multiple associations between cerebral metabolic disturbances and patient outcomes after moderate-to-severe TBI but marked heterogeneity and low certainty of evidence prevent firm prognostic conclusions. Our review indicates that the available data are therefore insufficient to support the routine use of individual CMD analytes for prognostic stratification in clinical care. These findings do not diminish the established role of CMD as a real-time, individualized bedside monitor within multimodal neurocritical care; rather, they outline the current limits of its evidence base as a standalone long-term prognostic factor. Standardization of analyte thresholds, probe location, monitoring windows, and outcome definitions, together with adequately adjusted analyses, is needed before their prognostic value can be established.

## Detailed description of individual author contributions

**Cansu Rehber, MD –** Conceptualization, methodology, literature search, data extraction, analysis, drafting of the manuscript, and corresponding author responsibilities.

**Sara Turella, MD –** Data extraction, methodological input, critical revision of the manuscript.

**Wilco C. Peul, MD, PhD –** Study supervision, conceptual input, critical revision of the manuscript.

**Raimund Helbok, MD –** Study supervision, conceptual input, critical revision of the manuscript.

**Thomas *A. van* Essen, MD, PhD, MSc** – Methodological support, data interpretation, critical revision.

**Jeroen T.J.*M. van* Dijck, MD, PhD –** Study supervision, conceptual input, critical revision of the manuscript.

## Ethics declaration

The authors have NOT obtained informed consent from participants or their legal representatives, as no primary data was collected.

This study was performed in compliance with relevant laws, regulatory frameworks and guidelines where the research took place. Ethics committee approval was not required under relevant laws and institutional guidelines. It is a systematic review therefore no new patient data was obtained, previously published articles were evaluated.

## Authorship requirements

Each author contributed substantially to the conception or design of the work, acquisition or interpretation of data, drafting or revising the manuscript, approved the final version, and agrees to be accountable for all aspects of the work. The final manuscript has been reviewed and approved by all authors.

## Ethical guidelines and IRB approval

This study is a systematic review of previously published literature and did not involve new data collection from human participants. Therefore, Institutional Review Board (IRB) approval and informed consent were not required. The study was conducted in accordance with established ethical standards and guidelines for systematic reviews.

## Reporting checklist

This systematic review was conducted and reported in accordance with the guidelines. A PRISMA checklist has been completed and is submitted with the manuscript.

## Artificial intelligence statement

Artificial intelligence tools were used solely for minor grammar and language correction during manuscript preparation. No AI tools were used for data analysis, interpretation of results, or generation of scientific content.

## Funding

This systematic review has received funding from EU research and innovation programme HORIZON Europe 2021 under grant agreement 101057332 and by the Innovate UK Horizon Europe Guarantee Programme, UKRI Reference Number 10041120.

## Declaration of competing interest

The authors declare that they have no known competing financial interests or personal relationships that could have appeared to influence the work reported in this paper.
