# Supplementary material for: Association of cerebral microdialysis monitoring with clinical outcomes after traumatic brain injury; A systematic review
Source: Brain Spine. 2026 Sep 2;6:106659. doi: 10.1016/j.bas.2026.106659 (PMC13571572; doi:10.1016/j.bas.2026.106659)
Supplement: Supplementary file 1 [file mmc2.docx]

| Author (Year)  ***Appendix B. Risk of bias assessment for observational studies using QUIPS^[1]^ tool.*** | D1 | D2 | D3 | D4 | D5 | D6 | Overall |
| --- | --- | --- | --- | --- | --- | --- | --- |
| Chamoun et al. (2010) | Low | High | Low | Low | High | Moderate | High |
| Clausen et al. (2005-08) | Low | High | Low | Low | High | Moderate | High |
| Clausen et al. (2005-10) | Low | High | Low | Low | High | Moderate | High |
| Eiden et al. (2019) | Low | High | Low | Low | Moderate | Moderate | High |
| Goodman et al. (1999) | Low | Low | Low | Low | Moderate | Moderate | Low |
| Gopinath et al. (2000) | Low | Low | Low | Low | High | Moderate | Moderate |
| Gupta et al. (2017) | Low | Moderate | Low | Low | High | Moderate | High |
| Hägglund et al. (2022) | Low | Low | Low | Low | Low | Moderate | Low |
| Hejčl et al. (2012) | Low | Low | Low | Low | High | Moderate | Moderate |
| Hlatky et al. (2004) | Low | High | Low | Low | Moderate | Moderate | High |
| Ho et al. (2008) | Low | Low | Low | Low | High | Moderate | Moderate |
| Igarashi et al. (2017) | Low | High | Low | Low | Moderate | Moderate | High |
| Karathanou et al. (2011) | Low | Moderate | Low | Low | Moderate | Moderate | Moderate |
| Koura et al. (1998) | Low | High | Low | Low | High | High | High |
| Li et al. (2008) | Low | Moderate | Low | Low | Moderate | Moderate | Moderate |
| Low et al. (2009) | Low | High | Low | Low | High | High | High |
| Marini et al. (2020) | Low | Moderate | Low | Low | Moderate | Moderate | Moderate |
| Mazzeo et al. (2006) | Low | Low | Low | Low | Moderate | Moderate | Low |
| Nelson et al. (2011) | Low | Low | Low | Low | Moderate | Moderate | Low |
| Nordström et al. (2016) | Low | High | Low | Low | Moderate | High | High |
| Oddo et al. (2008) | Low | High | Low | Low | High | Moderate | High |
| Olivecrona et al. (2009) | Moderate | Low | Moderate | Low | High | Moderate | High |
| Parafarou et al. (2011) | Low | High | Low | Low | High | Moderate | High |
| Peerdeman et al. (2003) | Low | Low | Low | Low | High | Moderate | Moderate |
| Rajagopalan et al. (2022) | Low | Moderate | Low | Low | High | Moderate | High |
| Reinert et al. (2007) | Low | Low | Low | Low | High | High | High |
| Sanchez et al. (2013) | Low | High | Low | Low | High | Moderate | High |
| Sánchez-Porras et al. (2012) | Low | Moderate | Low | Low | High | Moderate | High |
| Stein et al. (2012) | Low | Low | Low | Low | Moderate | High | Moderate |
| Thelin et al. (2014) | Low | Moderate | Low | Low | Moderate | Moderate | Moderate |
| Vespa et al. (2003) | Low | Low | Low | Low | High | Moderate | Moderate |
| Vespa et al. (2006) | Low | Moderate | Low | Low | High | Moderate | High |
| Zauner et al. (1997) | Low | Moderate | Low | Low | High | Moderate | High |

References:

[1]	Hayden JA, van der Windt DA, Cartwright JL, Côté P, Bombardier C. Assessing bias in studies of prognostic factors. Ann Intern Med. 2013;158(4):280–6.
