# Supplementary material for: Association of cerebral microdialysis monitoring with clinical outcomes after traumatic brain injury; A systematic review
Source: Brain Spine. 2026 Sep 2;6:106659. doi: 10.1016/j.bas.2026.106659 (PMC13571572; doi:10.1016/j.bas.2026.106659)
Supplement: Supplementary file 3 [file mmc1.doc]

## Appendix 1. Full Search Strategy

***Niet in Leiden beschikbare artikelen kunnen aangevraagd worden via:*** [*https://fd24.formdesk.com/universiteitleiden/aanvraag-ibl-lumc*](https://fd24.formdesk.com/universiteitleiden/aanvraag-ibl-lumc)***.***

- ***Systematic Review-registratie bij*** [*https://www.crd.york.ac.uk/prospero/*](https://www.crd.york.ac.uk/prospero/)
- *Prisma:* [*https://www.equator-network.org/reporting-guidelines/prisma-s/*](https://www.equator-network.org/reporting-guidelines/prisma-s/)
- *Prisma Flowchart -* [*http://prisma-statement.org/prismastatement/flowdiagram.aspx*](http://prisma-statement.org/prismastatement/flowdiagram.aspx)

**Update 17-4-2026**

**Regular references - total: 533 references, from which 31 [18+13] new, sourced from:**

- PubMed: 381 - 15 new&unique
- Embase: 338 - 0 new&unique
- Web of Science: 425 - 13 new&unique [20-4-2026 - after temporary technical problem at Web of Science]
- Cochrane Library: 46 - 1 new&unique (xx trial register items)
- Emcare: *replaced by CINAHL as of 1-1-2025.*
- CINAHL: 75 - 2 new&unique
- Academic Search Premier: *access ceased as of 1-1-2026.*

**Meeting abstract references - total: 216 references, from which 20 new, sourced from:**

- Embase: 182 - 20 new&unique
- Web of Science: 44 - 0 new&unique [20-4-2026 *temporary technical problem at Web of Science*]
- Cochrane Library: 5 - 0 new&unique
- Academic Search Premier: *access ceased as of 1-1-2026.*
- CINAHL: 0

Regular references - total d.d. 4-7-2024: 502 references, sourced from:

- PubMed: 366
- Embase: 334 - 31 unique
- Web of Science: 381 - 74 unique
- Cochrane Library: 46 - 19 unique (16 trial register items)
- Emcare: 123 - 1 unique
- Academic Search Premier: 213 - 11 unique

Meeting abstract references - total d.d. 4-7-2024: 196 references, sourced from:

- Embase: 167 - 166 unique
- Web of Science: 43 - 27 unique
- Cochrane Library: 5 - 0 unique
- Academic Search Premier: 5 - 3 unique

-> EndNote

--> rayyan - <https://rayyan.ai/>

Vraag:

The research question is: Does cerebral microdialysis monitorization have an association with patient functional outcome in moderate and severe TBI?

Population: 18+ patients with moderate to severe TBI (GCS below 12)

Outcome: functional outcome which is Glasgow coma scale in 6 months

intervention: cerebral microdialysis and measured analytes

There is a systematic review from 2017 that has also covered this topic, the only difference is their outcome includes other things besides functional outcome. The article is: Zeiler FA, Thelin EP, Helmy A, Czosnyka M, Hutchinson PJA, Menon DK. A systematic review of cerebral microdialysis and outcomes in TBI: relationships to patient functional outcome, neurophysiologic measures, and tissue outcome. Acta Neurochir (Wien). 2017 Dec;159(12):2245-2273. doi: 10.1007/s00701-017-3338-2. Epub 2017 Oct 7. PMID: 28988334

Relevant articles:

1. Bolcha M, Hejcl A, Prochazka J, Huskova E, Sames M (2014) Elevated intracranial pressure and impaired brain metabolism correlate with fatal outcome after severe brain injury. J Neurotrauma 31(5):A10 -> meeting abstract, identifiable in Embase : 71400007.an
2. Timofeev, Ivan, et al. "Cerebral extracellular chemistry and outcome following traumatic brain injury: a microdialysis study of 223 patients." Brain 134.2 (2011): 484-494. PMID 21247930
3. Belli A, Sen J, Petzold A, Russo S, Kitchen N, Smith M (2008) Metabolic failure precedes intracranial pressure rises in traumatic brain injury: a microdialysis study. Acta Neurochir 150(5):461–470 PMID 18421408

21247930 18421408 28988334

Databases:

**PubMed**

<http://www.ncbi.nlm.nih.gov/pubmed?otool=leiden>

(("Brain Injuries, Traumatic"[Mesh] OR "traumatic brain injury"[tw] OR "traumatic brain injuries"[tw] OR "Brain Trauma"[tw] OR "Brain Traumas"[tw] OR "Traumatic Encephalopathies"[tw] OR "Traumatic Encephalopathy"[tw] OR "traumatic head injury"[tw] OR "traumatic head injuries"[tw] OR "Head Trauma"[tw] OR "Head Traumas"[tw] OR "traumatic cerebral injury"[tw] OR "traumatic cerebral injuries"[tw] OR "Cerebral Trauma"[tw] OR "Cerebral Traumas"[tw] OR "severe TBI"[tw] OR "severe TBIs"[tw] OR "moderate TBI"[tw] OR "moderate TBIs"[tw] OR "TBI"[tw] OR "TBIs"[tw] OR "traumatic brain injury"[title/abstract:~6] OR "traumatic brain injuries"[title/abstract:~6] OR "traumatic brain injured"[title/abstract:~6] OR "Brain Trauma"[title/abstract:~6] OR "Brain Traumas"[title/abstract:~6] OR "Traumatic Encephalopathies"[title/abstract:~6] OR "Traumatic Encephalopathy"[title/abstract:~6] OR "traumatic head injury"[title/abstract:~6] OR "traumatic head injuries"[title/abstract:~6] OR "Head Trauma"[title/abstract:~6] OR "Head Traumas"[title/abstract:~6] OR "traumatic cerebral injury"[title/abstract:~6] OR "traumatic cerebral injuries"[title/abstract:~6] OR "Cerebral Trauma"[title/abstract:~6] OR "Cerebral Traumas"[title/abstract:~6] OR "severe TBI"[title/abstract:~6] OR "severe TBIs"[title/abstract:~6] OR "moderate TBI"[title/abstract:~6] OR "moderate TBIs"[title/abstract:~6] OR "Brain Concussion"[tw] OR "Brain Contusion"[tw] OR "Brain Concussion"[title/abstract:~6] OR "Brain Contusion"[title/abstract:~6] OR "Brain Concussed"[title/abstract:~6] OR "Brain Contused"[title/abstract:~6]) AND ("Microdialysis"[Mesh] OR "Microdialysis"[tw] OR "microdialysate"[tw] OR "microdialysates"[tw] OR "Microdialy*"[tw] OR "Micro dialysis"[tw] OR "micro dialysate"[tw] OR "micro dialysates"[tw] OR "Micro dialy*"[tw] OR "CMD analytes"[title/abstract:~0] OR "CMD analyte"[title/abstract:~0] OR "MD Probe"[tw] OR "MD Probes"[tw]) NOT (("Infant"[mesh] OR "infant"[ti] OR "infants"[ti] OR "baby"[ti] OR "babies"[ti] OR "Child"[mesh] OR "child"[ti] OR "children"[ti] OR "Adolescent"[mesh] OR "adolescent"[ti] OR "adolescents"[ti]) NOT ("Adult"[mesh] OR "adult"[ti] OR "adults"[ti] OR "elderly"[ti])) NOT (("Animals"[mesh] OR "veterinary"[ti] OR "rabbit"[ti] OR "rabbits"[ti] OR "animal"[ti] OR "animals"[ti] OR "murine"[ti] OR "mouse"[ti] OR "mice"[ti] OR "rodent"[ti] OR "rodents"[ti] OR "rat"[ti] OR "rats"[ti] OR "pig"[ti] OR "pigs"[ti] OR "porcine"[ti] OR "horse"[ti] OR "horses"[ti] OR "equine"[ti] OR "cow"[ti] OR "cows"[ti] OR "bovine"[ti] OR "goat"[ti] OR "goats"[ti] OR "sheep"[ti] OR "ovine"[ti] OR "canine"[ti] OR "dog"[ti] OR "dogs"[ti] OR "feline"[ti] OR "cat"[ti] OR "cats"[ti]) NOT "Humans"[mesh]) AND english[la])

Archival info:

OR **"brain injury"[tw] OR "brain injuries"[tw] OR "head injury"[tw] OR "head injuries"[tw] OR "cerebral injury"[tw] OR "cerebral injuries"[tw]**

**Embase**

<http://ovidsp.ovid.com/ovidweb.cgi?T=JS&PAGE=main&MODE=ovid&D=oemezd>

((*"Traumatic Brain Injury"/ OR "traumatic brain injury".ti,ab OR "traumatic brain injuries".ti,ab OR "Brain Trauma".ti,ab OR "Brain Traumas".ti,ab OR "Traumatic Encephalopathies".ti,ab OR "Traumatic Encephalopathy".ti,ab OR "traumatic head injury".ti,ab OR "traumatic head injuries".ti,ab OR "Head Trauma".ti,ab OR "Head Traumas".ti,ab OR "traumatic cerebral injury".ti,ab OR "traumatic cerebral injuries".ti,ab OR "Cerebral Trauma".ti,ab OR "Cerebral Traumas".ti,ab OR "severe TBI".ti,ab OR "severe TBIs".ti,ab OR "moderate TBI".ti,ab OR "moderate TBIs".ti,ab OR "TBI".ti,ab OR "TBIs".ti,ab OR (("traumatic" ADJ6 "brain" ADJ6 "injury") OR ("traumatic" ADJ6 "brain" ADJ6 "injuries") OR ("traumatic" ADJ6 "brain" ADJ6 "injured") OR ("Brain" ADJ6 "Trauma") OR ("Brain" ADJ6 "Traumas") OR ("Traumatic" ADJ6 "Encephalopathies") OR ("Traumatic" ADJ6 "Encephalopathy") OR ("traumatic" ADJ6 "head" ADJ6 "injury") OR ("traumatic" ADJ6 "head" ADJ6 "injuries") OR ("Head" ADJ6 "Trauma") OR ("Head" ADJ6 "Traumas") OR ("traumatic" ADJ6 "cerebral" ADJ6 "injury") OR ("traumatic" ADJ6 "cerebral" ADJ6 "injuries") OR ("Cerebral" ADJ6 "Trauma") OR ("Cerebral" ADJ6 "Traumas") OR ("severe" ADJ6 "TBI") OR ("severe" ADJ6 "TBIs") OR ("moderate" ADJ6 "TBI") OR ("moderate" ADJ6 "TBIs")).ti,ab OR "Brain Concussion".ti,ab OR "Brain Contusion".ti,ab OR (("Brain" ADJ6 "Concussion") OR ("Brain" ADJ6 "Contusion") OR ("Brain" ADJ6 "Concussed") OR ("Brain" ADJ6 "Contused")).ti,ab) AND (*"Microdialysis"/ OR *"microdialysis catheter"/ OR *"microdialysis system"/ OR "Microdialysis".ti,ab OR "microdialysate".ti,ab OR "microdialysates".ti,ab OR "Microdialy*".ti,ab OR "Micro dialysis".ti,ab OR "micro dialysate".ti,ab OR "micro dialysates".ti,ab OR "Micro dialy*".ti,ab OR "CMD analytes".ti,ab OR "CMD analyte".ti,ab OR "MD Probe".ti,ab OR "MD Probes".ti,ab) NOT ((exp "Infant"/ OR "infant".ti OR "infants".ti OR "baby".ti OR "babies".ti OR exp "Child"/ OR "child".ti OR "children".ti OR exp "Adolescent"/ OR "adolescent".ti OR "adolescents".ti) NOT (exp "Adult"/ OR "adult".ti OR "adults".ti OR "elderly".ti)) NOT ((exp "Animals"/ OR "veterinary".ti OR "rabbit".ti OR "rabbits".ti OR "animal".ti OR "animals".ti OR "murine".ti OR "mouse".ti OR "mice".ti OR "rodent".ti OR "rodents".ti OR "rat".ti OR "rats".ti OR "pig".ti OR "pigs".ti OR "porcine".ti OR "horse".ti OR "horses".ti OR "equine".ti OR "cow".ti OR "cows".ti OR "bovine".ti OR "goat".ti OR "goats".ti OR "sheep".ti OR "ovine".ti OR "canine".ti OR "dog".ti OR "dogs".ti OR "feline".ti OR "cat".ti OR "cats".ti) NOT exp "Human"/) AND english.la)

- NOT conference review.pt
- NOT (conference review or conference abstract).pt
- AND (conference abstract).pt

**Web of Science**

<http://isiknowledge.com/wos>

((TI=("Traumatic Brain Injury" OR "traumatic brain injury" OR "traumatic brain injuries" OR "Brain Trauma" OR "Brain Traumas" OR "Traumatic Encephalopathies" OR "Traumatic Encephalopathy" OR "traumatic head injury" OR "traumatic head injuries" OR "Head Trauma" OR "Head Traumas" OR "traumatic cerebral injury" OR "traumatic cerebral injuries" OR "Cerebral Trauma" OR "Cerebral Traumas" OR "severe TBI" OR "severe TBIs" OR "moderate TBI" OR "moderate TBIs" OR "TBI" OR "TBIs" OR (("traumatic" NEAR/6 "brain" NEAR/6 "injury") OR ("traumatic" NEAR/6 "brain" NEAR/6 "injuries") OR ("traumatic" NEAR/6 "brain" NEAR/6 "injured") OR ("Brain" NEAR/6 "Trauma") OR ("Brain" NEAR/6 "Traumas") OR ("Traumatic" NEAR/6 "Encephalopathies") OR ("Traumatic" NEAR/6 "Encephalopathy") OR ("traumatic" NEAR/6 "head" NEAR/6 "injury") OR ("traumatic" NEAR/6 "head" NEAR/6 "injuries") OR ("Head" NEAR/6 "Trauma") OR ("Head" NEAR/6 "Traumas") OR ("traumatic" NEAR/6 "cerebral" NEAR/6 "injury") OR ("traumatic" NEAR/6 "cerebral" NEAR/6 "injuries") OR ("Cerebral" NEAR/6 "Trauma") OR ("Cerebral" NEAR/6 "Traumas") OR ("severe" NEAR/6 "TBI") OR ("severe" NEAR/6 "TBIs") OR ("moderate" NEAR/6 "TBI") OR ("moderate" NEAR/6 "TBIs")) OR "Brain Concussion" OR "Brain Contusion" OR (("Brain" NEAR/6 "Concussion") OR ("Brain" NEAR/6 "Contusion") OR ("Brain" NEAR/6 "Concussed") OR ("Brain" NEAR/6 "Contused"))) OR AK=("Traumatic Brain Injury" OR "traumatic brain injury" OR "traumatic brain injuries" OR "Brain Trauma" OR "Brain Traumas" OR "Traumatic Encephalopathies" OR "Traumatic Encephalopathy" OR "traumatic head injury" OR "traumatic head injuries" OR "Head Trauma" OR "Head Traumas" OR "traumatic cerebral injury" OR "traumatic cerebral injuries" OR "Cerebral Trauma" OR "Cerebral Traumas" OR "severe TBI" OR "severe TBIs" OR "moderate TBI" OR "moderate TBIs" OR "TBI" OR "TBIs" OR (("traumatic" NEAR/6 "brain" NEAR/6 "injury") OR ("traumatic" NEAR/6 "brain" NEAR/6 "injuries") OR ("traumatic" NEAR/6 "brain" NEAR/6 "injured") OR ("Brain" NEAR/6 "Trauma") OR ("Brain" NEAR/6 "Traumas") OR ("Traumatic" NEAR/6 "Encephalopathies") OR ("Traumatic" NEAR/6 "Encephalopathy") OR ("traumatic" NEAR/6 "head" NEAR/6 "injury") OR ("traumatic" NEAR/6 "head" NEAR/6 "injuries") OR ("Head" NEAR/6 "Trauma") OR ("Head" NEAR/6 "Traumas") OR ("traumatic" NEAR/6 "cerebral" NEAR/6 "injury") OR ("traumatic" NEAR/6 "cerebral" NEAR/6 "injuries") OR ("Cerebral" NEAR/6 "Trauma") OR ("Cerebral" NEAR/6 "Traumas") OR ("severe" NEAR/6 "TBI") OR ("severe" NEAR/6 "TBIs") OR ("moderate" NEAR/6 "TBI") OR ("moderate" NEAR/6 "TBIs")) OR "Brain Concussion" OR "Brain Contusion" OR (("Brain" NEAR/6 "Concussion") OR ("Brain" NEAR/6 "Contusion") OR ("Brain" NEAR/6 "Concussed") OR ("Brain" NEAR/6 "Contused"))) OR AB=("Traumatic Brain Injury" OR "traumatic brain injury" OR "traumatic brain injuries" OR "Brain Trauma" OR "Brain Traumas" OR "Traumatic Encephalopathies" OR "Traumatic Encephalopathy" OR "traumatic head injury" OR "traumatic head injuries" OR "Head Trauma" OR "Head Traumas" OR "traumatic cerebral injury" OR "traumatic cerebral injuries" OR "Cerebral Trauma" OR "Cerebral Traumas" OR "severe TBI" OR "severe TBIs" OR "moderate TBI" OR "moderate TBIs" OR "TBI" OR "TBIs" OR (("traumatic" NEAR/6 "brain" NEAR/6 "injury") OR ("traumatic" NEAR/6 "brain" NEAR/6 "injuries") OR ("traumatic" NEAR/6 "brain" NEAR/6 "injured") OR ("Brain" NEAR/6 "Trauma") OR ("Brain" NEAR/6 "Traumas") OR ("Traumatic" NEAR/6 "Encephalopathies") OR ("Traumatic" NEAR/6 "Encephalopathy") OR ("traumatic" NEAR/6 "head" NEAR/6 "injury") OR ("traumatic" NEAR/6 "head" NEAR/6 "injuries") OR ("Head" NEAR/6 "Trauma") OR ("Head" NEAR/6 "Traumas") OR ("traumatic" NEAR/6 "cerebral" NEAR/6 "injury") OR ("traumatic" NEAR/6 "cerebral" NEAR/6 "injuries") OR ("Cerebral" NEAR/6 "Trauma") OR ("Cerebral" NEAR/6 "Traumas") OR ("severe" NEAR/6 "TBI") OR ("severe" NEAR/6 "TBIs") OR ("moderate" NEAR/6 "TBI") OR ("moderate" NEAR/6 "TBIs")) OR "Brain Concussion" OR "Brain Contusion" OR (("Brain" NEAR/6 "Concussion") OR ("Brain" NEAR/6 "Contusion") OR ("Brain" NEAR/6 "Concussed") OR ("Brain" NEAR/6 "Contused")))) AND (TI=("Microdialysis" OR "microdialysis catheter" OR "microdialysis system" OR "Microdialysis" OR "microdialysate" OR "microdialysates" OR "Microdialy*" OR "Micro dialysis" OR "micro dialysate" OR "micro dialysates" OR "Micro dialy*" OR "CMD analytes" OR "CMD analyte" OR "MD Probe" OR "MD Probes") OR AK=("Microdialysis" OR "microdialysis catheter" OR "microdialysis system" OR "Microdialysis" OR "microdialysate" OR "microdialysates" OR "Microdialy*" OR "Micro dialysis" OR "micro dialysate" OR "micro dialysates" OR "Micro dialy*" OR "CMD analytes" OR "CMD analyte" OR "MD Probe" OR "MD Probes") OR AB=("Microdialysis" OR "microdialysis catheter" OR "microdialysis system" OR "Microdialysis" OR "microdialysate" OR "microdialysates" OR "Microdialy*" OR "Micro dialysis" OR "micro dialysate" OR "micro dialysates" OR "Micro dialy*" OR "CMD analytes" OR "CMD analyte" OR "MD Probe" OR "MD Probes")) NOT (TI=(("Infant" OR "infant" OR "infants" OR "baby" OR "babies" OR "Child" OR "child" OR "children" OR "Adolescent" OR "adolescent" OR "adolescents") NOT ("Adult" OR "adult" OR "adults" OR "elderly")) OR AK=(("Infant" OR "infant" OR "infants" OR "baby" OR "babies" OR "Child" OR "child" OR "children" OR "Adolescent" OR "adolescent" OR "adolescents") NOT ("Adult" OR "adult" OR "adults" OR "elderly"))) NOT (TI=("Animals" OR "veterinary" OR "rabbit" OR "rabbits" OR "animal" OR "animals" OR "murine" OR "mouse" OR "mice" OR "rodent" OR "rodents" OR "rat" OR "rats" OR "pig" OR "pigs" OR "porcine" OR "horse" OR "horses" OR "equine" OR "cow" OR "cows" OR "bovine" OR "goat" OR "goats" OR "sheep" OR "ovine" OR "canine" OR "dog" OR "dogs" OR "feline" OR "cat" OR "cats") OR AK=("Animals" OR "veterinary" OR "rabbit" OR "rabbits" OR "animal" OR "animals" OR "murine" OR "mouse" OR "mice" OR "rodent" OR "rodents" OR "rat" OR "rats" OR "pig" OR "pigs" OR "porcine" OR "horse" OR "horses" OR "equine" OR "cow" OR "cows" OR "bovine" OR "goat" OR "goats" OR "sheep" OR "ovine" OR "canine" OR "dog" OR "dogs" OR "feline" OR "cat" OR "cats")) AND LA=english)

**Cochrane Library**

<https://www.cochranelibrary.com/advanced-search/search-manager>

(("Traumatic Brain Injury" OR "traumatic brain injury" OR "traumatic brain injuries" OR "Brain Trauma" OR "Brain Traumas" OR "Traumatic Encephalopathies" OR "Traumatic Encephalopathy" OR "traumatic head injury" OR "traumatic head injuries" OR "Head Trauma" OR "Head Traumas" OR "traumatic cerebral injury" OR "traumatic cerebral injuries" OR "Cerebral Trauma" OR "Cerebral Traumas" OR "severe TBI" OR "severe TBIs" OR "moderate TBI" OR "moderate TBIs" OR "TBI" OR "TBIs" OR (("traumatic" NEAR/6 "brain" NEAR/6 "injury") OR ("traumatic" NEAR/6 "brain" NEAR/6 "injuries") OR ("traumatic" NEAR/6 "brain" NEAR/6 "injured") OR ("Brain" NEAR/6 "Trauma") OR ("Brain" NEAR/6 "Traumas") OR ("Traumatic" NEAR/6 "Encephalopathies") OR ("Traumatic" NEAR/6 "Encephalopathy") OR ("traumatic" NEAR/6 "head" NEAR/6 "injury") OR ("traumatic" NEAR/6 "head" NEAR/6 "injuries") OR ("Head" NEAR/6 "Trauma") OR ("Head" NEAR/6 "Traumas") OR ("traumatic" NEAR/6 "cerebral" NEAR/6 "injury") OR ("traumatic" NEAR/6 "cerebral" NEAR/6 "injuries") OR ("Cerebral" NEAR/6 "Trauma") OR ("Cerebral" NEAR/6 "Traumas") OR ("severe" NEAR/6 "TBI") OR ("severe" NEAR/6 "TBIs") OR ("moderate" NEAR/6 "TBI") OR ("moderate" NEAR/6 "TBIs")) OR "Brain Concussion" OR "Brain Contusion" OR (("Brain" NEAR/6 "Concussion") OR ("Brain" NEAR/6 "Contusion") OR ("Brain" NEAR/6 "Concussed") OR ("Brain" NEAR/6 "Contused"))):ti,ab,kw AND ("Microdialysis" OR "microdialysis catheter" OR "microdialysis system" OR "Microdialysis" OR "microdialysate" OR "microdialysates" OR "Microdialy*" OR "Micro dialysis" OR "micro dialysate" OR "micro dialysates" OR "Micro dialy*" OR "CMD analytes" OR "CMD analyte" OR "MD Probe" OR "MD Probes"):ti,ab,kw NOT (("Infant" OR "infant" OR "infants" OR "baby" OR "babies" OR "Child" OR "child" OR "children" OR "Adolescent" OR "adolescent" OR "adolescents") NOT ("Adult" OR "adult" OR "adults" OR "elderly")):ti)

AND LA=english

(conference abstract OR meeting abstract OR conference proceeding OR conference proceedings):pt

**Emcare** <http://ovidsp.ovid.com/ovidweb.cgi?T=JS&NEWS=n&CSC=Y&PAGE=main&D=emcr>

Access ceased as of 1-1-2025 - replaced by CINAHL.

**CINAHL**

<https://catalogue.leidenuniv.nl/view/action/uresolver.do?operation=resolveService&package_service_id=33137965460002711&institutionId=2711&customerId=2710&VE=true>

Limit to Academic Journals.

LA=english

((XB("Traumatic Brain Injury" OR "traumatic brain injury" OR "traumatic brain injuries" OR "Brain Trauma" OR "Brain Traumas" OR "Traumatic Encephalopathies" OR "Traumatic Encephalopathy" OR "traumatic head injury" OR "traumatic head injuries" OR "Head Trauma" OR "Head Traumas" OR "traumatic cerebral injury" OR "traumatic cerebral injuries" OR "Cerebral Trauma" OR "Cerebral Traumas" OR "severe TBI" OR "severe TBIs" OR "moderate TBI" OR "moderate TBIs" OR "TBI" OR "TBIs" OR (("traumatic" N6 "brain" N6 "injury") OR ("traumatic" N6 "brain" N6 "injuries") OR ("traumatic" N6 "brain" N6 "injured") OR ("Brain" N6 "Trauma") OR ("Brain" N6 "Traumas") OR ("Traumatic" N6 "Encephalopathies") OR ("Traumatic" N6 "Encephalopathy") OR ("traumatic" N6 "head" N6 "injury") OR ("traumatic" N6 "head" N6 "injuries") OR ("Head" N6 "Trauma") OR ("Head" N6 "Traumas") OR ("traumatic" N6 "cerebral" N6 "injury") OR ("traumatic" N6 "cerebral" N6 "injuries") OR ("Cerebral" N6 "Trauma") OR ("Cerebral" N6 "Traumas") OR ("severe" N6 "TBI") OR ("severe" N6 "TBIs") OR ("moderate" N6 "TBI") OR ("moderate" N6 "TBIs")) OR "Brain Concussion" OR "Brain Contusion" OR (("Brain" N6 "Concussion") OR ("Brain" N6 "Contusion") OR ("Brain" N6 "Concussed") OR ("Brain" N6 "Contused"))) OR SU("Traumatic Brain Injury" OR "traumatic brain injury" OR "traumatic brain injuries" OR "Brain Trauma" OR "Brain Traumas" OR "Traumatic Encephalopathies" OR "Traumatic Encephalopathy" OR "traumatic head injury" OR "traumatic head injuries" OR "Head Trauma" OR "Head Traumas" OR "traumatic cerebral injury" OR "traumatic cerebral injuries" OR "Cerebral Trauma" OR "Cerebral Traumas" OR "severe TBI" OR "severe TBIs" OR "moderate TBI" OR "moderate TBIs" OR "TBI" OR "TBIs" OR (("traumatic" N6 "brain" N6 "injury") OR ("traumatic" N6 "brain" N6 "injuries") OR ("traumatic" N6 "brain" N6 "injured") OR ("Brain" N6 "Trauma") OR ("Brain" N6 "Traumas") OR ("Traumatic" N6 "Encephalopathies") OR ("Traumatic" N6 "Encephalopathy") OR ("traumatic" N6 "head" N6 "injury") OR ("traumatic" N6 "head" N6 "injuries") OR ("Head" N6 "Trauma") OR ("Head" N6 "Traumas") OR ("traumatic" N6 "cerebral" N6 "injury") OR ("traumatic" N6 "cerebral" N6 "injuries") OR ("Cerebral" N6 "Trauma") OR ("Cerebral" N6 "Traumas") OR ("severe" N6 "TBI") OR ("severe" N6 "TBIs") OR ("moderate" N6 "TBI") OR ("moderate" N6 "TBIs")) OR "Brain Concussion" OR "Brain Contusion" OR (("Brain" N6 "Concussion") OR ("Brain" N6 "Contusion") OR ("Brain" N6 "Concussed") OR ("Brain" N6 "Contused"))) OR MW("Traumatic Brain Injury" OR "traumatic brain injury" OR "traumatic brain injuries" OR "Brain Trauma" OR "Brain Traumas" OR "Traumatic Encephalopathies" OR "Traumatic Encephalopathy" OR "traumatic head injury" OR "traumatic head injuries" OR "Head Trauma" OR "Head Traumas" OR "traumatic cerebral injury" OR "traumatic cerebral injuries" OR "Cerebral Trauma" OR "Cerebral Traumas" OR "severe TBI" OR "severe TBIs" OR "moderate TBI" OR "moderate TBIs" OR "TBI" OR "TBIs" OR (("traumatic" N6 "brain" N6 "injury") OR ("traumatic" N6 "brain" N6 "injuries") OR ("traumatic" N6 "brain" N6 "injured") OR ("Brain" N6 "Trauma") OR ("Brain" N6 "Traumas") OR ("Traumatic" N6 "Encephalopathies") OR ("Traumatic" N6 "Encephalopathy") OR ("traumatic" N6 "head" N6 "injury") OR ("traumatic" N6 "head" N6 "injuries") OR ("Head" N6 "Trauma") OR ("Head" N6 "Traumas") OR ("traumatic" N6 "cerebral" N6 "injury") OR ("traumatic" N6 "cerebral" N6 "injuries") OR ("Cerebral" N6 "Trauma") OR ("Cerebral" N6 "Traumas") OR ("severe" N6 "TBI") OR ("severe" N6 "TBIs") OR ("moderate" N6 "TBI") OR ("moderate" N6 "TBIs")) OR "Brain Concussion" OR "Brain Contusion" OR (("Brain" N6 "Concussion") OR ("Brain" N6 "Contusion") OR ("Brain" N6 "Concussed") OR ("Brain" N6 "Contused")))) AND (XB("Microdialysis" OR "microdialysis catheter" OR "microdialysis system" OR "Microdialysis" OR "microdialysate" OR "microdialysates" OR "Microdialy*" OR "Micro dialysis" OR "micro dialysate" OR "micro dialysates" OR "Micro dialy*" OR "CMD analytes" OR "CMD analyte" OR "MD Probe" OR "MD Probes") OR SU("Microdialysis" OR "microdialysis catheter" OR "microdialysis system" OR "Microdialysis" OR "microdialysate" OR "microdialysates" OR "Microdialy*" OR "Micro dialysis" OR "micro dialysate" OR "micro dialysates" OR "Micro dialy*" OR "CMD analytes" OR "CMD analyte" OR "MD Probe" OR "MD Probes") OR MW("Microdialysis" OR "microdialysis catheter" OR "microdialysis system" OR "Microdialysis" OR "microdialysate" OR "microdialysates" OR "Microdialy*" OR "Micro dialysis" OR "micro dialysate" OR "micro dialysates" OR "Micro dialy*" OR "CMD analytes" OR "CMD analyte" OR "MD Probe" OR "MD Probes")) NOT TI(("Infant" OR "infant" OR "infants" OR "baby" OR "babies" OR "Child" OR "child" OR "children" OR "Adolescent" OR "adolescent" OR "adolescents") NOT ("Adult" OR "adult" OR "adults" OR "elderly")) NOT TI("Animals" OR "veterinary" OR "rabbit" OR "rabbits" OR "animal" OR "animals" OR "murine" OR "mouse" OR "mice" OR "rodent" OR "rodents" OR "rat" OR "rats" OR "pig" OR "pigs" OR "porcine" OR "horse" OR "horses" OR "equine" OR "cow" OR "cows" OR "bovine" OR "goat" OR "goats" OR "sheep" OR "ovine" OR "canine" OR "dog" OR "dogs" OR "feline" OR "cat" OR "cats")))

**Academic Search Premier**

<http://search.ebscohost.com/login.aspx?authtype=ip,uid&profile=lumc&defaultdb=aph>

Access ceased as of 1-1-2026.
